# Research on the Application of Biomimetic Design in Art and Design

**DOI:** 10.3390/biomimetics10080541

**Published:** 2025-08-18

**Authors:** Congrong Xiao, Dongkwon Seong

**Affiliations:** Department of X-Cultural Studies, Graduate School, Kookmin University, Seoul 02707, Republic of Korea; xiaocongrong@baiyunu.edu.cn

**Keywords:** biomimetic design, biological inspiration, art design, plant biomimetics, animal biomimetics, ecological biomimetics, sustainable design

## Abstract

Biomimetic design, derived from the study of biological systems, has emerged as a pivotal methodology in contemporary art and design. By systematically integrating the morphological traits, structural principles, and functional mechanisms of living organisms into design thinking, it provides both a novel theoretical perspective and methodological support for modern design practice. This design philosophy draws abundant inspiration from nature’s aesthetics and achieves a profound fusion of organic form and artistic expression. This study systematically traces the theoretical evolution of biomimetic design—from its early phase of direct form-mimicry to today’s holistic, systems-based approach—and clarifies its interdisciplinary logic and developmental trajectory. We examine its applications in public installations, product development, architecture, and fashion. Through a structured analysis of plant-inspired, animal-inspired, and ecosystem-inspired strategies—linked with the aesthetic demands and cultural contexts of design—this study uncovers the underlying mechanisms by which biological models drive innovation. The findings demonstrate that, by organically combining form simulation, function optimization, and ecological awareness, biomimetic design not only elevates the aesthetic value, visual impact, and emotional resonance of design works but also amplifies their social role and cultural significance. Moreover, its interdisciplinary potential in materials innovation, technological integration, and environmental sustainability highlights unique pathways for addressing complex contemporary challenges. This study adopts a methodology that blends case-study analysis and theoretical interpretation. Through an in-depth examination of exemplar projects, it validates that biomimetic design not only achieves a seamless unity of function and form but also offers a robust theoretical framework and practical strategies for sustainable design implementation. These insights advance both the theoretical depth and practical innovation of the design discipline.

## 1. Introduction

### 1.1. The Relationship Among Biomimetic Design, Art Design, and Design

Biomimetics emerged in the twentieth century as an interdisciplinary science born of rapid advances in biology and the technological sciences. Since its inception, it has demonstrated lasting vitality and relentless innovation, swiftly extending into numerous domains of the natural, technical, and engineering sciences while profoundly influencing the humanities and social sciences. This discipline, defined as the emulation of biological systems to create or enhance artificial systems, has evolved into a vital force for innovation across diverse domains.

At its core, biomimetic design involves systematically studying and applying the remarkable forms, structures, functions, materials, and behavioral strategies honed by organisms over millions of years of evolution. By analyzing natural phenomena—such as color, structural configuration, and acoustic patterns—designers translate these principles into concrete design solutions, enabling them to transcend conventional formal invention, instead embracing deep insight into evolutionary processes and their artistic transformation. Rather than merely replicating superficial shapes, biomimetic design engages with the functional and structural mechanisms of biological systems and the “hyper-rational” principles of natural efficiency and adaptability.

As an integrative methodology that unites scientific rigor and artistic sensibility, biomimetic design draws its theoretical foundation from the broader discipline of biomimetics, defined as “the science of imitating the principles of biological systems in constructing technical systems, or endowing artificial systems with features analogous to those of living organisms.” [1].

The impulse to imitate nature dates back to humanity’s earliest decorative arts. Primitive tools—wooden clubs and stone axes—directly mimicked the functional morphology of animal horns and claws; bone needles mirror the form of fish bones; and boat hulls derive from observations of fish bodies. In architecture, the Doric order of ancient Greece mirrors the sturdy physique of the mature male human form, whereas the Ionic order evokes the slender grace of the matured female silhouette. In ancient China, everyday vessels—such as tiger-shaped jars, zoomorphic ewers, eagle-shaped teapots, and ox-form lamps—and garment motifs like tortoise shell or fish scale patterns not only carried ritual or symbolic meaning (e.g., fertility, talismanic protection, or totemic worship) but also exemplified the transformation of real biological forms into decorative design elements [1,2].

Before the formal establishment of art and design as academic disciplines, decorative arts and art design existed in a state of natural unity. During the Renaissance, Giorgio Vasari introduced the concept of Arti del disegno, encompassing painting, sculpture, and architecture under a single rubric of “design,” thereby recognizing decorative arts as an intrinsic component of art design. Crafts of the period—pottery and bronze ornamentation, for instance—combined utility with decoration, making ornamentation the primary means of artistic expression and reflecting an original synthesis of function and aesthetics. Although later art history often relegated decorative arts to the status of “minor arts” or “crafts,” distinct from the “major arts” of painting and sculpture, their essential roles in beautifying objects, communicating meaning, and embodying the spirit of an era remained indispensable dimensions of design activity [3].

The late nineteenth-century Arts and Crafts movement, led by William Morris, marked a pivotal reevaluation of decorative art’s status. Reacting against the inferior quality engendered by industrial mass production, the movement championed “truth to materials” and the social responsibility of the designer—views that profoundly reshaped subsequent understandings of ornament’s value. Throughout history, natural flora and fauna have long served as fertile sources for decorative motifs. Owen Jones, in his seminal (1856), explicitly asserted that the forms found in nature constitute the fundamental resources of ornament design—a perspective that resonates strongly with the foundational tenets of contemporary biomimetic design [2].

The “Art Nouveau Movement” of the late 19th and early 20th centuries marked a pinnacle in the development of decorative arts, characterized by its veneration of the vital force inherent in nature and its endeavor to embody this vitality in creative expression. Designers within the movement extensively employed exaggerated, sinuous organic linear patterns and motifs, emulating the morphological characteristics of climbing vines and plants, thereby manifesting vigorous upward vitality. Notable exemplars include Victor Horta’s residential design at 12 Rue de Turin in Brussels, and Casa Milà and Sagrada Família in Barcelona, all of which epitomized the integration of natural forms into architectural design through decorative language. Antoni Gaudí, a devoted advocate of nature, regarded nature as the most perfect designer. His works were replete with biomimetic curves and organic forms, establishing the foundation for modern biomimetic design in both morphological language and emotional expression [3].

The early 20th century witnessed the emergence of the Bauhaus school, whose core principle of “form follows function” marked a paradigmatic shift in modern art and design theory. The principle of “unity of art and technology,” advocated by Walter Gropius, positioned traditional decorative arts as impediments to functional realization, thereby catalyzing a revolution toward design de-ornamentation. This ideological transformation manifested in fundamental changes to design language: geometric forms and standardized production supplanted elaborate ornamentation, exemplified by Marcel Breuer’s Wassily Chair as a quintessential representative of functionalist design. As design disciplines separated from fine arts and developed independently, decorative arts were relegated to the category of “minor arts,” while emerging disciplines such as industrial design and visual communication became the mainstream of modern design education. The biomimetic focus in design shifted from superficial morphological decoration to the emulation of organisms’ intrinsic mechanisms of high efficiency, low consumption, structural rationality, and functional superiority. This functional biomimicry emphasized addressing practical problems and enhancing product performance, reflecting a fundamental transition from “decoration for decoration’s sake” to “form expressing function.” [3].

In the manifestation of formal aesthetic principles, biomimetic design and decorative arts demonstrate remarkable commonalities. Whether through formal principles such as symmetry, rhythm, and gradation, or visual elements like proportion, color, and texture, perfect exemplars can be found within natural biological forms. The geometric order embodied in plant structures such as lotus seed pods and sunflowers not only inspired the functional design of biomimetic products but also provided a rich formal vocabulary for decorative arts. This concrete or abstract treatment of natural forms constituted both an important technique in decorative arts and the nascent form of early biomimetic design [4].

Contemporary design exhibits pluralistic tendencies, with biomimetic design and decorative arts achieving profound integration within new contexts. This synthesis transcends simple morphological imitation, placing greater emphasis on conveying nature’s emotional resonance, cultural symbols, and ecological principles. Biomimetic design imbues products with natural charm, symbolic vitality, and affinity, satisfying contemporary humanity’s emotional yearning for a return to nature. The biomimetic forms themselves possess inherent decorative qualities and emotional value. In contrast, decorative artistic techniques are employed to express the deeper implications and cultural identity of biomimetic designs, as exemplified in bamboo-inspired cup designs that embody profound Eastern cultural heritage [3].

With the transition of design philosophy from pure functionalist aesthetics toward humanistic and ecologically-oriented approaches, biomimetic design has gradually emerged as a significant methodology in contemporary design. It effectively resolves the dichotomy between form and function that plagued traditional design, thereby achieving genuine organic unity between form and function [3]. Biomimetic art design represents a comprehensive manifestation of purposiveness, not merely constituting formal beauty but also possessing profound theoretical foundations and technical underpinnings. This comprehensive characteristic enables biomimetic design to carry deeper cultural connotations and spiritual pursuits while fulfilling practical functions [5].

The integration of biomimetic design and decorative arts not only manifests at the visual formal level but also embodies profound cultural values and symbolic significance. It reflects humanity’s reverence for and learning attitude toward the natural world, representing the concrete practice of the traditional Chinese philosophical concept of “unity of heaven and humanity” in modern design [6]. Within the context of globalization, biomimetic design serves as a universal design language capable of transcending cultural boundaries to convey humanity’s shared ecological consciousness and aesthetic pursuits [3]. Simultaneously, biomimetic design practices under different cultural backgrounds also reflect the unique natural concepts and aesthetic traditions of various peoples, promoting the pluralistic development of design culture. More importantly, the philosophical outlook of “learning from nature” and “harmonious coexistence” embodied in biomimetic design aligns closely with contemporary green design and sustainable design principles. Emulating the operational principles and material characteristics of natural ecosystems not only constitutes decoration or form but also represents an important pathway toward environmental friendliness and resource conservation, transforming design itself into a form of “ecological decoration.”

According to the theoretical frameworks of modern design, design development should embody the unity of science, technology, and art. Biomimetic design, guided by this principle, has achieved systematic transcendence and innovative development of traditional decorative arts. Traditional decorative arts often remained at the level of superficial imitation and decorative application of natural forms, whereas biomimetic design penetrates deeper into the structural principles, functional mechanisms, and material characteristics of biological systems, fundamentally transforming from “formal resemblance” to “spiritual resemblance” and from “decorative function” to “functional utility.”

Traditional decorative arts’ appropriation of biological forms remained predominantly at the level of concrete imitation or abstract symbolism, as exemplified by the widespread circulation of decorative elements such as scrolling grass patterns, honeysuckle patterns, and lotus patterns. Contemporary designers frequently draw inspiration from natural forms such as bird nests and lotus flowers, creating designs that possess both decorative aesthetic appeal and functional satisfaction through the analysis and reconstruction of their structural characteristics. This transformative process transcends simple morphological imitation, constituting profound excavation and artistic sublimation of natural aesthetic principles [5].

The application of biomimetic design in contemporary art and design extends far beyond functional-level imitation, encompassing multiple artistic dimensions, including cultural connotation, emotional expression, and aesthetic experience. Designers imbue their works with unique aesthetic characteristics and cultural connotations through artistic refinement of animal and plant morphology and texture. Japanese designer Issey Miyake’s integration of shell textures and the layered sensibility of wood ear mushrooms into women’s fashion design fully demonstrates the artistic expressiveness of biomimetic design in the fashion domain. Across various categories, including packaging design, architectural design, and product design, biomimetic design presents rich artistic forms with the capacity for “artistic recreation of natural imagery,” enabling design works to carry profound emotional value, cultural connotation, and aesthetic significance while fulfilling functional requirements [5,7].

From early morphological decorative appropriation, through the Art Nouveau movement’s ultimate veneration of natural motifs, to the rise of functional biomimicry during the modernist period, and finally to the profound contemporary integration across emotional, symbolic, and sustainable dimensions, biomimetic design and decorative arts have continuously influenced and permeated each other. Biomimetic design both inherits the traditional focus on natural forms from decorative arts and achieves innovation through modern scientific and technological means, rendering it an extremely vital and promising methodology in contemporary art and design [8].

Biomimetic design provides decorative arts with an inexhaustible source of inspiration and scientific foundation, while decorative arts endow biomimetic designs with aesthetic value, emotional warmth, and cultural depth. Within the modern design context, “biomimicry” has become more than merely a means of achieving function or beautifying form; it has evolved into a design philosophy and aesthetic paradigm that connects humanity with nature, technology with the humanities, and the past with the future. It not only constructs morphology and structure through natural language but also endows design works with the threefold value of natural philosophy, aesthetic experience, and technological efficiency, representing the profound integration of design aesthetics and biological wisdom, thereby opening new pathways for the development of modern art and design.

### 1.2. The Status of Bionics Research in China and Internationally

The concept of “natural philosophy” has deep historical roots in China; however, the introduction and development of biomimetics as a modern discipline occurred relatively late. In 1960, the United States Air Force Aviation Agency first convened a biomimetics conference, where Steele proposed the concept of “bionics,” a term derived from Greek, meaning “the science of studying life systems.” Subsequently, industry and research domains began to strongly emphasize the theoretical construction and practical application of biomimetics. Early research was primarily concentrated in the field of industrial design, particularly in aircraft and vehicle design, providing innovative solutions for achieving high-efficiency and energy-saving objectives. Renowned designer Luigi Colani proposed a design philosophy inspired by nature, emphasizing the enhancement of products’ functionality and aesthetic value through the emulation of natural structures and morphologies. Colani’s “human-centered design” concept advanced the development of biomimetic design, enabling it to transcend the simple imitation of nature and evolve into an innovative design methodology [4].

In 1963, the term “bionics” was translated as “FangShengXue” (biomimicry, literally “imitating biology”) in Chinese, and systematic biomimetics research commenced around 1964. Subsequently, biomimetics reached several significant milestones: in January 1976, the Chinese Academy of Sciences successfully hosted China’s first biomimetics symposium in Beijing; in 1977, China’s biomimetics research plan was formally established at the “National Natural Science Discipline Planning Conference”; in 1979, the China Institute of Intelligence was established with biomimetic perception and intelligent technology as its primary research directions; in December 2003, an academic symposium titled “The Scientific Significance and Frontiers of Bionics” was convened; in 2010, the International Society of Bionic Engineering was formally established [1].

Regarding theoretical research on the developmental stages of biomimetics, scholars have proposed various classification models. In 2004, Yang divided the historical development of biomimetic design into five stages: prehistoric, ancient Chinese, ancient foreign, modern, and contemporary, providing a reference framework for tracing the historical trajectory of biomimetic design. In 2007, Lu Yongxiang, former President of the Chinese Academy of Sciences and President of Zhejiang University, pointed out that the biological world serves as a continuous source of human learning and that emulating the natural world possesses infinite potential for enhancing human creativity. In the same year, Wang proposed that “biomimetic design thinking originates from the combination of biomimetics and creative thinking,” exploring pathways for cultivating students’ biomimetic design thinking in industrial design education by integrating theory and practice. In 2008, Jing summarized the history of biomimetic design into three developmental stages: direct biomimicry (perceptual biomimicry), indirect biomimicry (rational biomimicry), and comprehensive biomimicry (scientific biomimicry), reflecting the historical inevitable trend of design development from ancient to contemporary periods [9].

In the realm of theoretical research and methodological construction, scholars have conducted in-depth exploration. In 2004, Yu summarized five core characteristics of biomimetic design science, systematically introducing biomimetic design from six dimensions, including color biomimicry, structural biomimicry, and morphological biomimicry, while proposing research pathways for biological models and technological models. In 2007, Ding employed cognitive psychology and product semantics theory to interpret product morphological biomimetic design, proposing a design process of “biological morphological observation and cognition–simplification–product design transformation.” During the same period, Dai analyzed biomimetic morphology, color, and texture in product design, constructing a comprehensive research framework from demand analysis to design evaluation [10].

In 2012, Gao proposed a philosophically elevated viewpoint, arguing that one should “develop systematic and macroscopic cognition of nature in practice, incorporating humanity into the ‘natural’ system” and pointing out that Laozi’s concept of “unity of heaven and humanity” enables design practice to connect with philosophy, with “biomimicry” functioning both as a methodology for morphological creation and as a holistic guiding principle. In 2013, Yu systematically discussed the philosophy, reasoning, and methods of biomimetic design, foreseeing that biomimetic design would profoundly influence people’s daily lives and values [10].

**Scheme 1:**
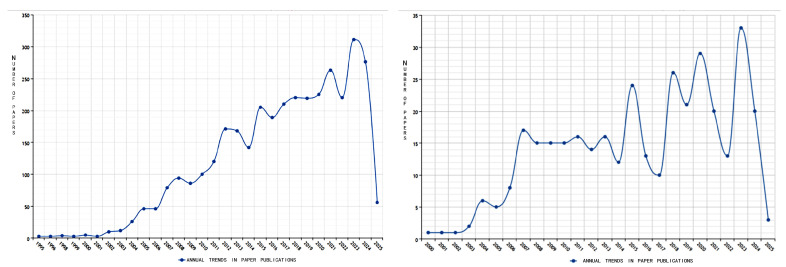
Annual publication quantity and trends of biomimetic design and biomimetic product-related papers in CNKI from left to right.

**Scheme 2:**
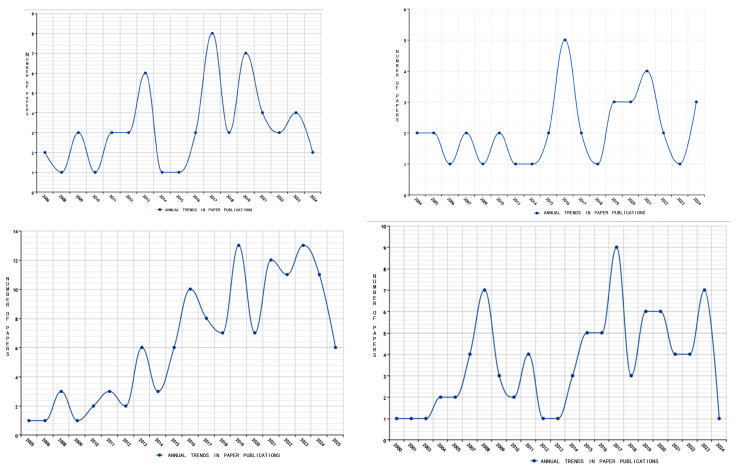
Annual publication quantity and trends of biomimetic clothing, biomimetic art, biomimetic concepts, and biomimetic notion papers in CNKI from left to right, top to bottom, respectively.

**Scheme 3:**
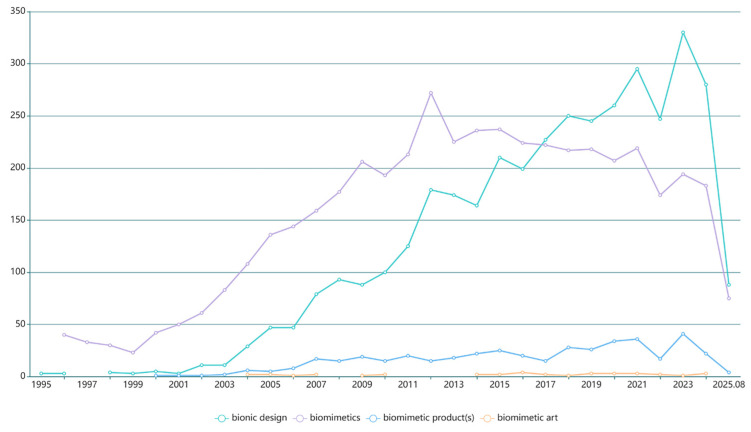
Comparison of paper publication trends for “Bionic Design,” “Biomimetics,” “Biomimetic Product(s),” and “Biomimetic Art” in CNKI.

According to the statistical data from the China National Knowledge Infrastructure (CNKI), as of August 2025, there are a total of 3592 papers related to “bionic design,” including 246 doctoral dissertations and 1196 master’s theses. The temporal distribution of publication volume, as shown in Scheme 1, indicates a continuous growth in public demand for bionic product design and its applications, driven by advancements in technology and improvements in living standards. However, the data presented in Scheme 2 reveals a relative scarcity in research concerning bionic theory (philosophy) and its artistic dimensions. For instance, searches using the keywords “bionic philosophy” and “bionic art” show that from 2004 to July 2025, 113 and 30 related papers were published, respectively. This reflects a gradual increase in attention to bionic philosophy over the past two decades, with the research field expanding from the initial focus on biology and mechanical engineering to include art and design disciplines. A comparative analysis of the publication trends related to “bionic design,” “biomimetics,” “bionic products,” and “bionic art” is shown in Scheme 3.

Among the highly cited literature, Zhang (cited 292 times) focuses on design theory and control strategies for flexible exoskeleton systems, while Luo et al. (cited 177 times) systematically elaborate on the developmental status of six major domains—morphological biomimicry, functional biomimicry, structural biomimicry, textural biomimicry, color biomimicry, and conceptual biomimicry—providing theoretical guidance and methodological support for biomimetic design.

The field of biomimetic research has experienced remarkable growth and institutional development over recent decades, with numerous internationally renowned higher education institutions actively participating in both theoretical research and practical applications. Among these, the Center for Biomimetic Biology at the University of California, Berkeley, has achieved particularly noteworthy results and enjoys a prestigious reputation in the international academic community. As research has deepened and diversified, influential biomimetic works have successively shaped the field’s theoretical foundations, establishing it as a rigorous interdisciplinary field.

Contemporary biomimetic research demonstrates remarkable diversity and sophistication, as evidenced by recent comprehensive reviews and original research contributions. Reviews such as that by Rosellini et al. have advanced our understanding of scaffold design that mimics the layered structure of natural vascular walls, while Cui et al.’s analysis has elucidated the complex coordination mechanisms in biological and artificial ciliary systems. Original research has produced equally impressive innovations, including Fang et al.’s investigation of tail dynamics in gecko-inspired robots, Casagualda et al.’s development of mussel-inspired multifunctional polyethylene glycol nanoparticle interfaces using catechol-based adhesion strategies, and Ehrlich et al.’s groundbreaking discovery of silactins in sponge biosilica structures through desilicified analysis. Additional notable contributions include Winand et al.’s TriTrap robotic gripper inspired by insect tarsal chains, Chen et al.’s capillary wicking surfaces mimicking Heliamphora minor trichomes, and Saint-Sardos et al.’s development of the taxonomy-driven “Bioinspire-Explore” tool for biodiversity data analysis. These diverse research endeavors collectively demonstrate how biomimetic principles are being systematically applied across disciplines, from robotics and materials science to fluid dynamics and biotechnology, establishing biomimetics as a cornerstone of contemporary innovation in engineering and biological sciences [11].

Systematically examining the developmental trajectory and research status of biomimetic design both China and internationally shows that this field is currently at a critical stage of profound integration from theoretical construction to practical application, possessing broad developmental prospects and significant practical significance.

Statistical data reveal that although thousands of papers related to “biomimetic design” have been published in China and internationally, literature concerning “biomimetic art” remains extremely limited, indicating a notable deficiency in biomimetics research within the art and design domain. International research has primarily focused on specific technical applications, such as biological materials, robotics technology, and medical applications. For instance, the latest articles published in the journal *Biomimetics* are primarily concentrated in biomimetic mechanisms and design, biomimetic robotics, biofabrication and characterization, biomimetic and bioinspired chemistry, bioinspired sensing, nanotribology, nanomechanics, micro/nanoscale studies on biological systems, biomimetics inspired by animal and plant biomechanics, synthetic and biohybrid systems, self-organization and cooperative behavior, biomimetic aspects of tissue engineering, and bioinspired, biomedical and biomolecular materials, among other core research areas. However, the present study takes a distinctive approach, focusing specifically on applications within the art and design domain. Unlike existing research that emphasizes technical functionality, this study infuses biomimetic design with cultural and artistic connotations, thereby exploring how biomimetics can achieve both “imitating nature” and “creating aesthetic appeal” in art and design. This approach not only expands the application boundaries of biomimetics but also provides the art and design field with innovative creative approaches and methodologies, extending its scope from traditional engineering and technical applications into the realm of artistic creation.

## 2. The Concept and Application of Biomimetic Art Design

The German philosopher Hegel proposed that “beauty is the sensuous manifestation of the idea,” thus laying the theoretical foundation for design aesthetics. Designing according to the principles of beauty allows the creation of works with profound aesthetic significance. In this context, “beauty” encompasses not only the visual aspect of attractiveness but also the functional aspect of usability. In the design field, a truly “beautiful” product should meet users’ needs to the greatest extent and achieve an organic unity of form and function [9].

As a design methodology that integrates scientific rationality and artistic sensibility, biomimetic design has its aesthetic theory rooted in a deep understanding of and innovative application to the aesthetic laws found in nature. This theoretical system is based on profound insights into natural laws and artistic reconstruction, achieved through systematic imitation of biological forms, structural mechanisms, functional processes, and cultural imagery. Ultimately, it strives for the harmonious unity of “human–nature–machine.” The value of biomimetic design aesthetics is reflected not only in the surface imitation of biological forms but also in the abstract extraction and innovative reconstruction of natural aesthetic principles, resulting in a unique and comprehensive aesthetic system.

Biomimetic design goes beyond the decorative aspects of traditional design by effectively addressing functional pain points through structural biomimicry. Functional aesthetics theory asserts that true beauty is not merely found in the visual presentation of form but is embodied in the perfect realization of function. This theory emphasizes the harmonious unity of form and function, deeply embodying the classical aesthetic principle that “form follows function.”

Biomimetic design can be categorized into several directions, such as morphological biomimetics, functional biomimetics, structural biomimetics, color biomimetics, texture biomimetics, and cultural biomimetics, which is more inclined toward the arts. It also includes material biomimetics, a technical implementation method for structural or functional biomimetics. The specific carriers of these categories (morphology, function, structure, etc.) can be divided into animal biomimetics, plant biomimetics, and ecological biomimetics. Naturally, these fields often overlap in practical design because biological organisms are unified in form–function–structure, necessitating multidimensional coordination in biomimetic design [12].

Morphological biomimetics constitutes the core component of biomimetic design aesthetics. Biological forms in nature have evolved over millions of years under selective pressures, forming structures that are highly functional while maintaining visual appeal [9]. Biomimetic design uses curves as the aesthetic vehicle, effectively overcoming the mechanical feel of geometric straight lines and imbuing products with organic vitality. From an aesthetic typology perspective, geometric curves (such as spheres and cones) embody the aesthetic characteristics of rational order, while free curves (such as biological textures) convey the aesthetic connotations of dynamic emotions.

The design philosophy of “there are no straight lines in the universe,” proposed by the German designer Luigi Colani, forms an important theoretical foundation for modern biomimetic design. This theory emphasizes the representation of natural laws through curvilinear forms, arguing that organic curved surfaces are more aligned with the principles of natural morphogenesis and ergonomics. By employing continuous curvature transition technology, Colani successfully replicated the harmonious features of biological organic forms in his iconic egg-shaped tea set design, providing a classic example for morphological bionics. This theoretical framework not only reflects a profound understanding of natural aesthetic laws but also establishes a solid philosophical foundation for biomimetic practices in industrial design. In the design of consumer electronic products, Colani successfully applied his biomimetic philosophy to the development process of the Canon T90 camera. This design replaced the traditional linear structure with a streamlined surface atop the pentaprism, simulating the mechanical characteristics of insect exoskeletons, thereby significantly enhancing human-machine interaction comfort. The product’s casing employs parametric surface design, optimizing hand fit through precise surface transitions and effectively reducing muscle fatigue during prolonged use. This practice demonstrated the considerable advantages of curvilinear forms in ergonomic applications, providing valuable practical guidance for subsequent biomimetic designs in electronic products [5,13].

The morphological biomimetic design in the automotive industry primarily draws upon the low-drag physiological structure of sharks as the core reference model. Such designs feature elements such as longitudinally extended hoods, low-profile front fascias, and continuous contour surfaces, all of which are directly inspired by the hydrodynamic morphology of a shark’s head. From the perspective of design development, the “Shark Nose” design pioneered by BMW models in the 1960s and 1970s utilized a forward-tilting grille to effectively reduce the drag coefficient. The Ferrari F430 employed a gill-slit bionic structure for its air intakes, optimizing the airflow path for cooling. Similarly, the side vents of the Hyundai Kia Genesis Coupe achieved the design goal of form-function integration. The BMW G20 and subsequent generations of the 3 Series have reintroduced the early “Shark Nose” design style, reflecting the metabolic continuity of bionic design genes. This design revival not only embodies the era-specific characteristics of biomimetic aesthetics but also validates the enduring value of natural forms in industrial design. A comprehensive analysis reveals that the core value of morphological biomimicry design is evident in three key areas: at the biomimicry level, curved shapes better align with the natural morphological principles of form generation; at the functionalism level, surface designs optimize mechanical properties and physical field distributions; and at the human-centered design level, organic curves significantly enhance physiological adaptability. Future research could explore the application potential of emerging technologies, such as geometrically adjustable surfaces, to further advance the use of lightweight, high-strength structures across disciplines [5,13]. Textural biomimicry, as a significant branch of biomimetic design, has opened innovative domains that emphasize functional and aesthetic values in contemporary art and design through precise simulation of surface textures, tactile qualities, and structural characteristics of biological organisms or natural objects. This design methodology not only achieves breakthroughs in visual effects but also demonstrates unique value across dimensions such as materials science, technological craftsmanship, and user experience.

In the realm of fashion and textile design, the application of textural biomimicry has evolved from simple visual imitation to a sophisticated system of technological innovation. McQueen’s 2010 reptilian texture collection represents a classic achievement in this domain. The collection created striking visual effects by simulating the tactile texture and iridescent luster of ancient reptilian scales. The designer employed digital drawing techniques to precisely convert biological dermal textures into print patterns, combining laser cutting and three-dimensional sewing techniques to recreate the complex layering of snake and lizard skins on leather and textile materials.

The development of synthetic fur and bark-textured fabrics has further expanded the application scope of textural biomimicry in textiles. Leopard print and snakeskin fabrics, through the organic integration of jacquard weaving and printing technologies, precisely simulate the patterns and tactile sensations of animal fur, providing innovative solutions for luxury fashion design that balance ethical considerations with visual effects. This technological development not only satisfies consumers’ pursuit of aesthetic quality but also reflects the design industry’s positive response to and practice of animal protection principles.

Biomimetic materials, through appropriation and simulation of structural characteristics, functional mechanisms, and formation pathways developed by natural organisms during prolonged evolutionary processes, drive the design and fabrication of novel intelligent materials. This approach not only directly utilizes the superior properties of natural materials (such as shells and spider silk) but also transforms complex biological microstructures into controllable artificial synthetic systems through biomimetic design. With natural components as the foundation, it integrates sustainable development concepts and achieves organic coupling of functions, including high strength, self-cleaning, and self-adaptation. Designers draw inspiration from biological morphology, structural functions, and color textures in nature, employing advanced technologies such as digital simulation, parametric modeling, and additive manufacturing to endow works with an organic unity of functionality and aesthetics. This fusion not only embodies reverence for and inheritance of natural wisdom but also demonstrates the profound alignment between sustainable development and innovative aesthetics, providing new creative paradigms and practical pathways for modern products, architecture, and public art [5].

The Silk Pavilion by Neri Oxman pioneers the “collaborative biomimicry” paradigm by incorporating living organisms (silkworms) into the manufacturing process, marking a philosophical shift in biomimetic design from “imitation” to “symbiosis.” The Silk Pavilion is a groundbreaking interdisciplinary project launched in 2013 by the Media Lab at the Massachusetts Institute of Technology (MIT), led by Oxman’s Mediated Matter Group. This project is based on the concept of Material Ecology, which introduces a trans-species design experiment integrating artificial construction with natural ecology. Oxman’s concept emphasizes that design should emulate the growth logic of natural systems rather than adhering to traditional mechanical assembly methods. The pavilion’s core philosophy is “growth over assembly,” viewing the silkworm as a “biological printer,” where its natural silk-spinning behavior contributes to the generation of building materials and the formation of architectural shapes. Through this design, the project facilitates an organic collaboration between human technology and natural organisms, exploring the integration of biological processes and algorithms in architectural manufacturing, thus transcending traditional design and production methods. Technologically, the pavilion relies on the organic integration of digital algorithms and biological fabrication. The preliminary structure consists of 26 polygonal silk panels, with the silk thread paths precisely positioned using CNC machines to create the basic framework. Through algorithmic control, silk distribution is varied based on the silkworms’ natural behavior, guiding the direction and density of silk production. Specifically, 6500 silkworms were placed on the supporting structure, where they autonomously selected silk-spinning areas based on environmental variables such as light and temperature. Over the course of 15 to 20 days, the silkworms filled in the voids predefined by the algorithm, resulting in a two-layered structure. The lower layer is a rigid framework woven by robots, while the upper layer consists of the organic fiber network generated by the silkworms’ silk. In this way, through biological fabrication, the pavilion achieved the transformation “from biological behavior to architectural material,” demonstrating the potential of biologically generated architecture [14].

The success of the Silk Pavilion project has provided both theoretical and practical foundations for subsequent research and technological development. In 2020, commissioned by the Museum of Modern Art (MoMA) in New York, the team developed the Silk Pavilion II project, upgrading the system to a dynamic loom configuration. By utilizing a rotating spindle to guide the silkworms in vertical silk production, the team successfully constructed a 6-m-high dynamic structure. This project further explored the interaction between gravity and biological behavior, offering significant insights for innovative applications across fields such as architecture, biomaterials, fashion, and aerospace. Additionally, the project inspired the development of biological 3D printing technologies and collaborative robotic construction systems, such as the Aguahoja biodegradable biomaterials and the Fiberbots swarm robotics system. These advancements have contributed to the cross-disciplinary application of materials and have played a pivotal role in driving a material revolution.

The integration of biomimetic materials with artistic design centers on appropriating nature’s “form” (morphology/texture), borrowing nature’s “force” (structure/function), and conveying nature’s “spirit” (ecological concepts/emotional resonance). Typical cases address environmental concerns while creating artistic experiences that combine scientific rigor with poetic sensibility through waste material regeneration (wood chip ceramics), biological structure transformation (leaf veins and vertebrae), or intelligent responsive design (thermochromic color-changing materials), thereby advancing design toward the evolution of “coexistence with nature.” [15].

Through mimicking the chromatic patterns and coloration mechanisms of organisms in nature, biomimetic coloration enables designers to create works that possess visual aesthetic appeal and functional richness, forming distinctive designs across multiple domains, including product packaging, spatial environments, and fashion brands.

The formation of biological color primarily relies on three natural mechanisms, providing the scientific foundation for biomimetic coloration technologies. The first is structural color biomimicry, whose principle lies in organisms generating colors through the interference, diffraction, or scattering of light waves by microstructures (such as photonic crystals, multilayer films, and diffraction gratings), rather than relying on chemical pigments. Typical examples include the iridescent effects of peacock feathers and the metallic luster of morpho butterfly wings. In biomimetic applications, this principle is employed in designing environmentally friendly materials that require no dyes, such as anti-counterfeiting labels and dynamic displays. The second is pigmentary color biomimicry, based on the mechanism whereby pigments within organisms (such as melanin and carotenoids) produce color through the absorption of specific wavelengths of light, exemplified by the chameleon’s color-changing ability through the contraction and expansion of pigment cells. Corresponding biomimetic applications include thermochromic coatings and emotion-responsive textiles. The third type is bioluminescent biomimicry, whose principle involves organisms converting chemical energy into light energy through biochemical reactions, producing cold light effects, such as the luciferase reaction in fireflies. This mechanism has inspired the development of biocompatible light sources and highly sensitive biosensors [16].

Based on the functional roles of biological color in nature, biomimetic design has established a clear functional classification system. Camouflage and defense functions originate from biological survival strategies, such as the octopus adjusting its pigment cells to simulate environmental textures, and the leaf butterfly’s wing coloration changing with environmental conditions. This function translates into dynamic adaptability in military camouflage uniforms and environmental coordination in architectural facade coatings within design applications. Information transmission and attraction functions are manifested in natural phenomena such as the vivid colors of flowers attracting pollinators and the feather displays of birds during courtship. Design applications include the use of saturated colors in children’s product packaging and the mimicry of bright poison frog coloration in warning signage. Environmental adaptation functions reflect intelligent biological responses to environmental conditions, such as the light-colored fur of camels reflecting sunlight for cooling and the bioluminescence of deep-sea creatures to lure prey. In the realm of product and packaging design, corresponding design practices include mimicking the gradient effects of natural coloration in children’s food packaging to enhance consumer appetite and trust; luxury anti-counterfeiting labels that utilize the optical properties of structural color to provide security assurance that is difficult to replicate. In architecture and materials science, smart windows achieve dynamic regulation of light transmission through electrochromic technology, while sports stadium roofs employ thermochromic materials to reduce energy consumption [17].

The core of functional biomimicry lies in the systematic transformation of biological adaptive mechanisms in nature into design language through in-depth research. This transformation process is not merely simple replication or imitation, but rather a profound understanding and innovative application of natural laws, fully embodying the effective integration of scientific rigor and artistic expression within biomimetic design aesthetic theory. Designers not only effectively solve complex problems in the real world but also imbue their works with profound aesthetic value and emotional resonance. This design philosophy transcends traditional functionalist frameworks, transforming the wisdom accumulated through millions of years of natural evolution into innovative solutions for human life, while simultaneously awakening people’s deep perceptual and emotional connection to natural aesthetics.

Structural biomimicry refers to a design methodology that systematically studies the internal structural characteristics of organisms (including skeletal systems, tissue textures, cellular arrangements, etc.), extracts their mechanical principles and organizational logic, and transforms these into engineering and technological solutions. Its core lies in simulating the performance advantages of biological structures, such as lightweight characteristics, strength–toughness properties, and energy efficiency, rather than merely remaining at the level of superficial morphological replication.

The invention of Velcro represents a landmark in the commercialization of biomimetic design, marking a breakthrough in the transition from natural observation to product innovation. The term “Velcro” is a portmanteau of the French words velour (velvet) and crochet (hook), created in 1948 by Swiss engineer George de Mestral, whose inspiration was drawn from the adhesive mechanism of burdock seeds. Mestral proposed artificially replicating the “hook-and-loop” structure of the burdock seed: one side was designed as a rigid material with hooks, while the other side consisted of soft, looped fibers, enabling rapid attachment and detachment. The initial samples were made from cotton, but their susceptibility to wear led to a shift toward nylon and polyester fibers, improving durability [18]. In the aerospace sector, further advancements involved the use of Teflon rings and polyester hooks, making the material suitable for extreme environments. Velcro gained widespread recognition when NASA utilized it to secure tools during space missions. Today, it is extensively applied in a variety of industries, including fashion (as an alternative to shoelaces), medical (bandages), aerospace (tool fixation in spacesuits), and automotive (seat covers) sectors [14].

The design of the Japanese Shinkansen train’s nose is a classic example of the successful application of structural biomimicry in the transportation sector, representing the first instance of biomimicry being applied to large-scale transportation infrastructure. In the 1990s, Japanese engineer Eiji Nakatsu obtained design inspiration by observing the kingfisher’s specialized ability to dive into water for fishing while addressing the technical challenge of “tunnel boom” generated by the Shinkansen train during high-speed travel. The unique structure of the kingfisher’s slender, conical beak enables it to penetrate water with virtually no splash, thereby minimizing water flow resistance. Based on this biomimetic principle, the new nose design adopted a graduated longitudinal section structure to buffer airflow, replacing the original bullet-shaped nose design, successfully achieving the technical objective of “silent acceleration.” This design not only realized optimization at the functional level but also presented elegant, streamlined aesthetics at the visual level, fully demonstrating that curved surface aesthetics possesses the dual characteristics of technical rationality and natural dynamism [14].

The similarities, differences, or overlapping aspects between structural biomimicry and “morphological biomimicry” (focusing on aesthetic symbolic expression) and “functional biomimicry” (simulating biological behavioral mechanisms) lie in the fact that structural biomimicry concentrates on the engineering translation of physical construction, emphasizing technical breakthroughs through deep-level structural analysis. The design logic of structural biomimicry is manifested in the following key aspects:

The first is multi-scale structural analysis. The scale transition from micro to macro constitutes an important characteristic of structural biomimicry. For example, the “brick-mortar” layered structure of shells (microscopic level) has been successfully applied to increase the toughness of bulletproof ceramics; the hexagonal structure of honeycombs (macroscopic level) is widely applied in the development of lightweight materials for spacecraft.

**Figure 1:**
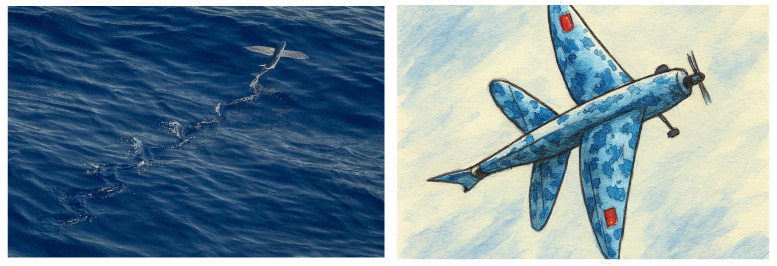
Flying fish in the wild (author: John Cobb, image source: unsplash.com) and drawing of the flying fish drone by the Shen Haijun team (author: Congrong Xiao).

The second is topological optimization and mechanical efficiency. Through topological analysis of biological structures, optimal material distribution is achieved. For instance, the skeletal distribution characteristics of flying fish pectoral fins inspired the carbon fiber structural design of unmanned aerial vehicle wing ribs, achieving ultra-high performance with a lift-to-drag ratio of 25. For example, the flying fish-inspired unmanned aerial vehicle designed by Professor Shen Haijun and his team from the School of Aerospace Engineering and Applied Mechanics at Tongji University as an example (Figure 1) features a wingspan of 1.5 m, body length of 1.8 m, tricycle landing gear configuration, and high-efficiency twin-blade propellers, powered by a high-power electric motor and 6S lithium battery.

The primary technical challenges facing structural biomimicry include the necessity for interdisciplinary knowledge integration, involving the comprehensive application of biological anatomy, materials mechanics, and computer simulation technologies (such as computational fluid dynamics analysis and finite element simulation), while simultaneously requiring extremely high manufacturing precision, such as 3D printing technologies for micrometer-scale porous structures.

Structural biomimicry not only plays a significant role in the engineering and technological domains but also demonstrates unique value in the artistic design field. Through in-depth research and transformation of biological structures, structural biomimicry provides contemporary design practice with dual pathways of technological innovation and aesthetic expression, propelling design toward more intelligent, ecological, and humanistic directions of development.

In addition to the aforementioned biomimetic designs related to natural organisms, the aesthetic theory of biomimetic design carries profound cultural and artistic implications. This connotation extends beyond merely external imitation of natural forms, representing instead an innovative practice that integrates specific human cultural symbols and traditions with natural design—this is cultural biomimicry—transforming biological imagery into cultural symbols and effectively conveying national spirit and philosophical essence.

Regarding the relationship between cultural biomimicry and biomimetic design, two primary perspectives exist within academic discourse. The first perspective considers cultural biomimicry as a new form of biomimetic design. Scholars such as Song define cultural biomimicry as “the integration of traditional cultural design and biomimetic design” [19], achieving biomimicry through the functional, structural, and morphological aspects of cultural biomimetic objects (natural objects, artifacts, and cultural symbols imbued with cultural significance), thereby embodying the cultural connotations of these cultural biomimetic objects.

The second perspective regards cultural biomimicry as a subset of symbolic biomimicry within morphological biomimicry. Scholars such as Fang contend that cultural biomimicry employs biomimetic thinking to extract forms from entities in the cultural domain, engaging in innovative integration with product forms, behaviors, and contexts. They define cultural biomimetic objects as encompassing entities from the cultural domain that extend beyond natural phenomena. From the perspective of product morphological biomimetic design, scholars define symbolic biomimicry as the extraction of biological characteristics and their symbolic meanings [19].

Regarding the focus of cultural biomimicry, both perspectives concentrate on the cultural domain, yet the second perspective is limited to attention on the morphological characteristics of culture. From the perspective of cultural spatiality, mid-layer culture centered on human behavioral activities can embody cultural connotations through dynamic behavioral characteristics. Compared to the second perspective, the first perspective provides a more comprehensive analysis of cultural biomimicry, advocating for multidimensional attention to cultural presentation methods encompassing form, function, structure, and other dimensions. Consequently, two approaches emerge in defining cultural biomimetic objects: the first defines them as entities from the cultural domain; the second defines them as natural organisms possessing cultural significance [6].

**Figure 2:**
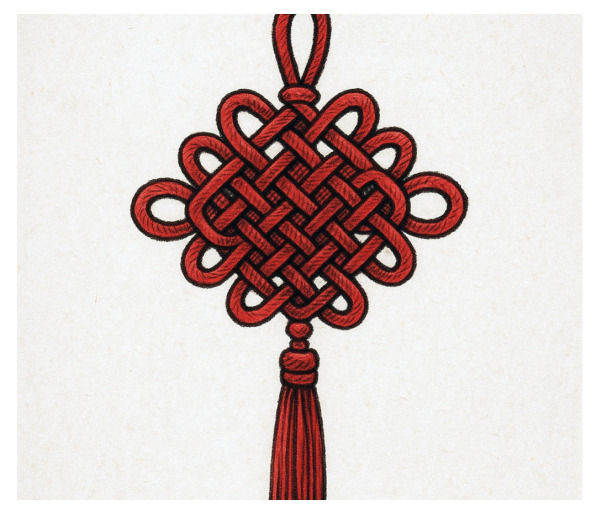
Drawing of Chinese knot (author: Congrong Xiao).

Taking the China Unicom logo as an example, its core element derives from the Buddhist “Panchang pattern” (also known as the “Chinese knot”) (Figure 2), whose interlocking and interconnected linear structure symbolizes the infinite connectivity and information flow of communication networks, aligning with the philosophical concepts of Eastern cyclical cosmology. The English design of “China Unicom” also incorporates the connotations of dual “i” (representing “I” (individual user) and “Information”), embodying the service philosophy of “customer-centricity” and reinforcing the symbiotic relationship between tradition and modernity [9].

**Figure 3:**
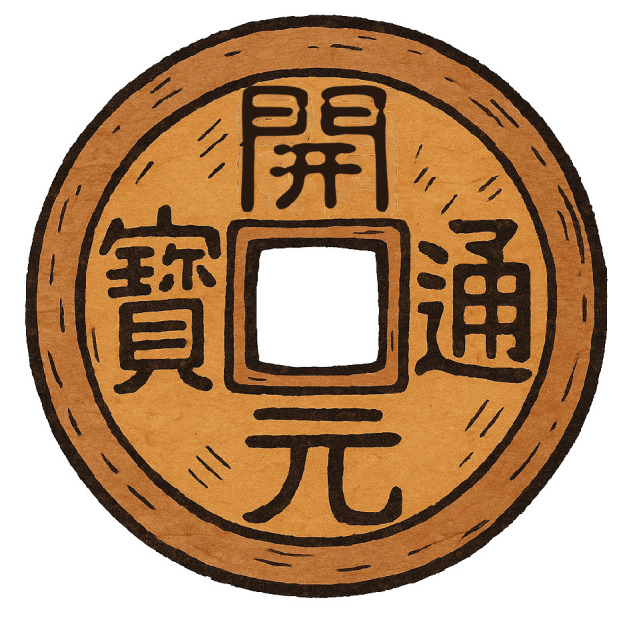
Drawing of a copper coin “Kaiyuan Tongbao” (author: Congrong Xiao).

In contrast, the Bank of China logo is prototyped after ancient Chinese copper coins (Figure 3), with its outer circle and inner square symbolizing the traditional cosmological view that “Heaven is round, Earth is square”. The central square aperture is transformed into the Chinese character “Zhong” (Central), simultaneously referencing “Bank of China” and implying the financial ethics of “centrality and harmony”. This circular contour conveys stability and trustworthiness, while the square aperture reinforces a sense of order, aligning with the attribute requirements of the banking industry. This design elevates the physical form of currency into a cultural symbol, achieving unity between function and aesthetics [9].

In addition to the aforementioned common fields of biomimetic design, the aesthetic theory of contemporary biomimetic design also profoundly embodies important concepts such as ecological aesthetics. This concept emphasizes the harmonious coexistence of design with the natural environment, pursuing aesthetic values aligned with sustainable development. Ecological aesthetics posits that true beauty should harmonize with the natural environment, satisfying human needs while preserving the ecological balance of nature, thus achieving the harmonious development of humanity and nature.

Several design works by Luigi Colani fully demonstrate the integration of multidisciplinary fields, creating innovative products that possess both aesthetic value and functional requirements. Colani explicitly articulated his design philosophy: “What I do is simply imitate the truths that nature reveals to us.” This design philosophy profoundly embodies the core idea of ecological aesthetics: by emulating the wisdom of nature, he creates design works that are both beautiful and environmentally friendly. In the early 2000s, Colani was invited to participate in the overall conceptual design of the Shanghai Chongming Island Ecological Science and Technology City. Centered on “biodynamic” principles, he proposed an urban morphological concept that simulated the structure of the human torso, emphasizing a design philosophy that combined “unity of heaven and humanity” with sustainable development. This urban planning proposal adopted a biomimetic structure of a “reclining female human body”: left hand (airport), right hand (harbor), feet (information center), breasts (shopping district), heart (energy center), and lungs (sanatorium). Colani chose the female body because “earth and femininity belong to yin,” aligning with the Chinese concept of yin-yang harmony, and planned to design a complementary “male city” to achieve yin-yang balance. The design planning of Chongming City precisely positioned architectural clusters according to meridian pathways, thereby simulating energy flow between the human body and the city. This design mapped the physiological functions of human organs (such as cardiac energy supply and pulmonary respiration) onto the sustainable operations of urban systems (energy production and air purification), fully embodying the core concept of “ecological science and technology city.” Although this concept was ultimately not implemented due to multiple factors, including technology, funding, and policy, its “biomimetic organic” appearance and integrated renewable energy system framework have profoundly influenced the design and ecological planning fields [5].

With technological advancement, the artistic application of biomimetic design is evolving toward intelligent aesthetics. Intelligent aesthetics emphasizes that design works should not only possess static visual appeal but also dynamic interactive aesthetic characteristics. Through the integration of advanced technologies such as artificial intelligence and sensing technology, biomimetic design can create aesthetic works with adaptive and intelligent features. This developmental trend indicates that the aesthetic theory of biomimetic design is progressing from traditional morphological imitation toward higher levels, such as functional biomimicry and behavioral biomimicry, reflecting the profound integration of aesthetic theory and technological progress.

In summary, the artistic theory and application of biomimetic design constitute a multidimensional, multi-layered theoretical system that not only encompasses the recognition and application of natural aesthetic laws but also embodies the unity of multiple values, including science, art, culture, and ecology. Biomimetic aesthetics achieves the four-dimensional unity of function, form, psychology, and culture: it follows the rational principle of “form follows function” (such as the fluid dynamics optimization of the Shinkansen), while awakening emotional resonance through curved language and natural symbols (such as the life metaphor in oval teapots), and constructs a symbiotic philosophy of “human–nature–machine” at the cultural level. This provides abundant aesthetic resources and innovative ideas for contemporary art design, promoting the development and advancement of design aesthetics. Biomimetic design will continue to penetrate interdisciplinary fields, using natural wisdom to drive the aesthetic revolution of “technological poeticization” and deeply integrate human design practices with natural laws.

## 3. Analysis of the Application of Biomimetic Design in the Field of Art and Design

### 3.1. Biomimetic Applications in Landscape Architecture Design

As an innovative design concept, biomimetic design has demonstrated enormous application potential and value in landscape architecture. In the contemporary context of pursuing ecological harmony, biomimetic design has become the core driving force in landscape creation, transcending mere references to natural elements and evolving into a systematic design logic infused with biological intelligence. Through in-depth observation and simulation of the morphological characteristics, structural features, and ecological functions of natural organisms, landscape designers can create landscape spaces that possess both artistic beauty and ecological wisdom.

#### 3.1.1. Morphological Biomimetics of Landscape Architecture Design

Morphological biomimetics is the most intuitive and widely applied approach in landscape architecture design. By replicating the shapes, textures, and structural characteristics of natural organisms, it creates landscape spaces that seamlessly integrate with the natural environment. This design strategy manifests not only in the overall creation of large-scale landscape structures but also deeply penetrates into the detailed treatment of landscape elements, forming a comprehensive biomimetic design system spanning from macro to micro levels.

Based on the degree of simulation and abstraction level, morphological biomimetics can be categorized into two primary types: concrete and abstract. Concrete biomimetics directly imitates the external morphological features of organisms, such as the concrete expression of lion forms and Buddhist imagery in the rockery of Suzhou Lion Grove Garden, the horticultural techniques of trimming plants into bird and animal shapes in residential landscape design, and the design of landscape elements like lotus leaf sunshades and petal-shaped rest pavilions [20]. Concrete biomimetics emphasizes visual affinity and direct expression of natural interest. Conversely, abstract biomimetics involves artistic reconstruction through extracting the essential characteristics of biological forms, such as the application of hexagonal honeycomb structures in paving or landscape constructions, which embodies geometric aesthetic characteristics while maintaining structural stability; the design of meandering, snake-like streamlined pathways in modern landscapes mimics the dynamic rhythm of natural water flows through abstract techniques [21].

The intricate structures of organisms provide rich, innovative inspiration for revolutionizing traditional landscape construction design. Biomimetic inspiration from plant structures has demonstrated significant advantages in modern landscape design. For instance, landscape pavilion designs inspired by the morphology of dawn redwood ingeniously transform layered branch structures into load-bearing framework systems, achieving excellent mechanical efficiency and structural stability while visually simulating the dynamic beauty of upward tree growth. The lightweight characteristics of bamboo’s hollow segmented structure have been widely applied in pergola design [22], achieving dual objectives of material conservation and structural optimization [23].

Biomimetic applications of animal structures are particularly prominent in technological innovation. The topological optimization principles of lightweight avian bones, combined with 3D printing technology, can construct hollow landscape structures (such as pavilions and observation platforms), achieving material reduction of over 30% while maintaining high-efficiency load-bearing performance. The geometric properties of hexagonal honeycomb close-packing structures are applied in parametric designs for sunshade canopies or ecological wall systems, integrating shading, ventilation, and aesthetic functions. The cable-net structure of the Munich Olympic Stadium in Germany originated from spider web biomimetic principles, demonstrating the successful application of biomimetic structures in large-scale architecture.

Modern landscape biomimetic design has transcended mere morphological simulation, evolving into a comprehensive biomimetic strategy that coordinates function and form. A notable case of ecological function integration is Chengdu Living Water Garden, which mimics the self-purification system of natural water bodies by constructing an ecological chain treatment process of anaerobic pools → plant beds → fish ponds, achieving ecological purification of wastewater and embodying the organic combination of functional and morphological biomimetics. Similarly, vertical forest apartments mimic the three-dimensional structure of forest ecosystems, achieving 1.2 kg CO_2_/m^2^ annual carbon sequestration [24].

Furthermore, modern digital technologies provide robust technical support for the precise implementation of complex biomimetic structures. 3D printing technology and BIM modeling systems enable the precise construction of complex geometric forms such as honeycomb topological configurations; parametric design tools enhance the design efficiency and manufacturing precision of biomimetic structures. The development of smart material technology further expands the application scope of biomimetic design, such as the use of adaptive materials and shape-memory alloys in landscape constructions, enabling dynamic structural responsiveness and functional optimization.

Biomimetic design achieves a high degree of unity between aesthetic expression and practical functionality. “Flower street paving” mimics plant textures to enhance the natural charm of landscapes, while honeycomb structure paving improves load-bearing performance and drainage efficiency. In terms of cultural integration, biomimetic design combines with regional cultural characteristics, such as dragon walls and moon gate designs, endowing modern landscapes with deeper cultural symbolic significance. With the ongoing development of biotechnology, smart materials, and digital manufacturing technologies, landscape biomimetic design is progressing toward more dynamic, functional, and sustainable directions, providing a broad innovative space and practical pathways for future landscape design [20].

#### 3.1.2. Functional Biomimetics of Landscape Architecture Design

Functional biomimetics represents the deep application of biomimetic design, with its essence lying in transcending superficial morphological simulation to deeply explore and apply the survival mechanisms and adaptive strategies of natural organisms. Modern landscape design systematically studies the excellent sustainable systems in nature, converting biological intelligence into practical solutions for landscape functions, achieving coordinated unity between ecological benefits and design aesthetics.

The branching network structure of plant root systems exhibits highly efficient functions for rainwater absorption, transmission, and distribution, providing valuable biomimetic inspiration for the design of landscape drainage systems. By performing in-depth analyses of the multi-level branching morphology of root systems, designers can construct scientifically sound and rational drainage networks that enhance the infiltration efficiency and distribution equilibrium of surface water in landscapes.

China’s “sponge city” concept reflects a profound borrowing and application of natural ecological rainwater management wisdom. Under China’s rapid urbanization, the proportion of hardened surfaces has significantly increased, with 70% to 80% of urban rainfall converting to surface runoff and only 20% to 30% of rainwater being infiltrated, resulting in a series of problems, including urban waterlogging, post-rainfall drought, and aquatic ecological degradation. To mitigate these issues, the “sponge city” concept, drawing on the layered structure of forest floor layers and natural soil infiltration mechanisms as models, integrates permeable paving, rain gardens, bioretention ponds, and natural wetlands to construct a multi-level, comprehensive rainwater management system. This system not only efficiently intercepts, purifies, and stores rainwater while alleviating flood pressure but also replenishes groundwater resources and strengthens the circulatory resilience of urban ecosystems [13,25].

The Chenghu Aquatic Eight Immortals Ecological Culture Park in Suzhou is an example of the “sponge city” concept in application. This project deeply explores the ecological functional value of traditional Jiangnan aquatic plants—water bamboo, lotus root, water celery, foxnut, arrowhead, water chestnut, watershield, and water caltrop (collectively known as “Aquatic Eight Immortals”). These plants not only carry the cultural aesthetics of Jiangnan water towns but also possess excellent water purification capabilities. The project team designed stepped ecological purification pond groups based on analyses of the spatial distribution patterns of wetland plant root systems. The project achieved efficient rainwater interception and biological degradation of pollutants, thereby significantly enhancing water body self-purification and recycling utilization levels.

Regarding biomimetic material technology, shell and bamboo node structures have inspired innovative applications in landscape building materials. Shells consist of 95% calcium carbonate and 5% protein adhesive arranged in alternating layers, demonstrating excellent tensile strength. Based on this biological arrangement, self-cleaning coatings and thin-shell structure technologies have successively emerged: the former are widely used on building surfaces to facilitate the automatic decomposition of surface contaminants; the latter include high-strength lightweight concrete in landscape pavilions, domes, and other components, simulating the arched load-bearing method of shells to reduce material usage while significantly enhancing wind pressure resistance. The hollow multi-chamber structure of bamboo nodes combines high toughness with low density, inspiring the development of honeycomb foam concrete that integrates thermal insulation and lightweight advantages. Similarly, composite pipes designed to mimic the orientation of bamboo node fiber demonstrate compressive strength improvements of over 30% compared to traditional pipes when applied in underground drainage systems [26].

The most extensive implementation of functional biomimetic design is evident in the simulation and reconstruction of collaborative mechanisms in natural ecological systems, achieving landscape sustainability goals through systematic biomimetic strategies. For example, concrete crack repair technology has been developed based on biological trauma response mechanisms, where microcapsules that contain healing agents are embedded into the concrete before it sets, achieving “self-healing” functions that significantly extend structural service life. Similarly, by simulating the multi-layered planting structure of tropical rainforests, the construction of three-dimensional vegetation community systems can significantly enhance ecosystem resilience and reduce artificial maintenance requirements. In aquatic landscape biomimetic design, constructing closed-loop ecological water purification systems by simulating the food chain structure of wetland ecosystems achieves natural water purification and dynamic maintenance of ecological balance while reducing external energy input. The comprehensive application of these functional biomimetic technologies not only enhances the ecological efficiency of landscape systems but also provides technical pathways and practical frameworks for sustainable landscape design.

#### 3.1.3. Ecosystem Biomimetics of Landscape Architecture Design

The application of biomimetic design in landscapes can be categorized into four progressive levels according to the depth of biomimetic implementation: morphological biomimetics (such as structures mimicking plant leaf forms), structural biomimetics (such as hexagonal honeycomb structures used in paving or construction), functional biomimetics (such as artificial wetlands mimicking water body self-purification functions), and ecosystem biomimetics. Among these, ecosystem biomimetics, as the highest level of biomimetic implementation, requires the integration of biological communities, energy flow, and material cycling to reconstruct ecological systems approaching natural conditions. Research strongly indicates that “the imitation and borrowing from ecosystems as a whole represents a higher stage of biomimetic content,” with its core lying in emulating the structural stability, interspecies symbiotic relationships, and energy cycling mechanisms of natural ecosystems [27].

The central objective of ecosystem biomimetics lies in achieving multiple goals by mimicking the spatial arrangements of natural ecosystems (such as vertically stratified vegetation), interspecies relationships (such as companion plant combinations), and material cycling pathways (such as carbon, water, and nutrient flows), including enhancing biodiversity, strengthening system anti-interference capacity, and reducing artificial maintenance requirements. Through this systematic approach, landscape design can effectively promote biodiversity conservation, reduce negative environmental impacts, and achieve harmonious coexistence between artificial landscapes and natural ecology [26,28].

The “Derivative Floating Wetland” project at Yunnan Lijiang Lashihai Wetland Park provides a conceptual framework for ecosystem biomimetic design. It aimed to address the severe challenges facing wetland ecosystems: in part due to tourism development, the wetland area in China has decreased by 12% (According to a research report from the Chinese Academy of Sciences, China’s total wetland area experienced a net reduction of 60,900 square kilometers over the past 40 years, representing a relative loss rate of 12%.), and water quality grade has declined from Class II to Class IV. The design team established a comprehensive, tripart design positioning of “ecological protection–science education–recreational touring,” employing systematic biomimetic strategies to construct a sustainable wetland ecosystem. The project relies on stream flow for water supplementation, which, after on-site purification treatment, ultimately flows into Lashihai Wetland, forming a diversified landscape ecosystem that integrates water quality purification, ecological conservation, and environmental education functions.

Based on ecological protection hierarchy concepts, the project employs a concentric spatial layout model. The core protection zone is dominated by natural substrates, achieving minimal- or zero-intervention management modes through display-type ecological installation design. The buffer restoration zone is configured with reversible ecological restoration installations and migratory bird observation facilities, achieving the progressive ecological restoration of damaged wetlands. The edge interaction zone deploys diversified interactive installations, constructing multi-dimensional interactive experience spaces through ecological education corridors, forming a functional gradient distribution pattern from interior to exterior.

**Figure 4:**
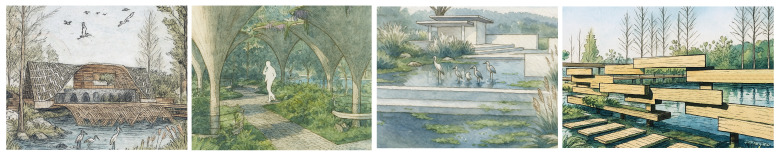
Drawings from left to right: dovetail, linfan, pick up time, wooden road (author: Congrong Xiao).

The project’s innovation centers on the design and application of four innovative biomimetic installations. The “Dovetail” (Figure 4) wetland resource detection device, inspired by migratory bird tail wings, employs streamlined double-bifurcated structures with main bodies constructed from lightweight carbon fiber and light-transmitting glass plate composites. Solar panels on the upper wing surface and water quality sensors and underwater camera equipment on the lower layer integrate morphological and functional biomimetics. The “Lin Fan” (Figure 4) rainwater collection device employs biomimetic funnel-shaped structures with porous bases to promote vegetation development and microbial habitation, enhancing natural surface runoff infiltration capacity through permeable paving and ecological grass planting systems. The “Pick Up Time” (Figure 4) ecological display device leverages multi-dimensional interactive technology to transform ecological knowledge into visualized educational scenarios, constructing immersive cognitive learning environments through five-sensory design. The “Wooden Road” (Figure 4) observation device features staggered concealed observation structures, requiring visitors to cooperatively push open the “wetland windows” by hand and view diverse wetland scenery through different landscape observation frames, guiding visitors to reflect on humanity’s dual role in ecosystem evolution.

#### 3.1.4. Summary of Biomimetic Applications in Landscape Architecture Design

Biomimetic design in landscape architecture transcends surface imitation, deeply integrating the efficient functionality, intelligent adaptability, and structural aesthetic principles of the natural world. Rather than simple mimicry of nature, it leverages a deep understanding of natural laws to create landscape environments that balance human with high ecological value.

Whether enhancing water cycle resilience through “sponge cities,” building lightweight ecological structures, or integrating intelligent responsive sustainable technologies, biomimetic design represents the core strategy for addressing contemporary ecological challenges and realizing the aesthetic vision of human–earth symbiosis. This design philosophy transcends the aesthetic orientation of traditional landscape design, integrating ecological wisdom, functional optimization, and artistic expression to provide new developmental directions for modern landscape architecture design.

Through biomimetic design, modern landscape architecture can not only satisfy aesthetic aspirations but also enable people to experience the beauty and vitality of nature within modernized environments, achieving a synthesis of technological progress and natural harmony. Future landscape architecture will continue to explore more advanced ecological solutions, advancing toward intelligent, sustainable organic systems and contributing to sustainable urban environments.

### 3.2. Biomimetic Art in Architectural Design

The application of biomimetic technology in architectural design represents a significant direction in the development of modern architecture, demonstrating the profound integration of science and aesthetics. Biomimetic architecture refers to the utilization of biomimetic principles, where the morphology, structure, and functions of organisms are mimicked to create architectural forms with biological characteristics in architectural design. As an emerging interdisciplinary science, biomimetics studies the structure and properties of biological systems, providing new design concepts and operational principles for the development of technology. Its application in architecture has enhanced the safety, economic efficiency, and environmental adaptability of buildings.

Biomimetic architecture encompasses three major domains: structural biomimetics, morphological biomimetics, and ecological functional biomimetics. Morphological biomimetics focuses on mimicking the external morphological characteristics of organisms to reflect biological aesthetic values in architecture; structural biomimetics emphasizes learning from the internal structural principles of organisms and applying them to architectural structural design; functional biomimetics stresses mimicking the functional characteristics of organisms, enabling buildings to possess adaptability and responsiveness similar to organisms [5].

From the perspective of biomimetic objects, biomimetic architecture is primarily divided into animal biomimetic architecture and plant biomimetic architecture. The former creates architectural forms with dynamic aesthetic appeal by mimicking the morphological structures of animals, such as bird wings and insect compound eyes. The latter draws from the growth patterns and structural characteristics of plants, such as tree branch structures and leaf textures. Biomimetic architectural structures primarily include fiber structures, bubble structures, spiral structures, skeletal structures, and shell structures.

#### 3.2.1. Structural Biomimetics of Architectural Design

**Figure 5:**
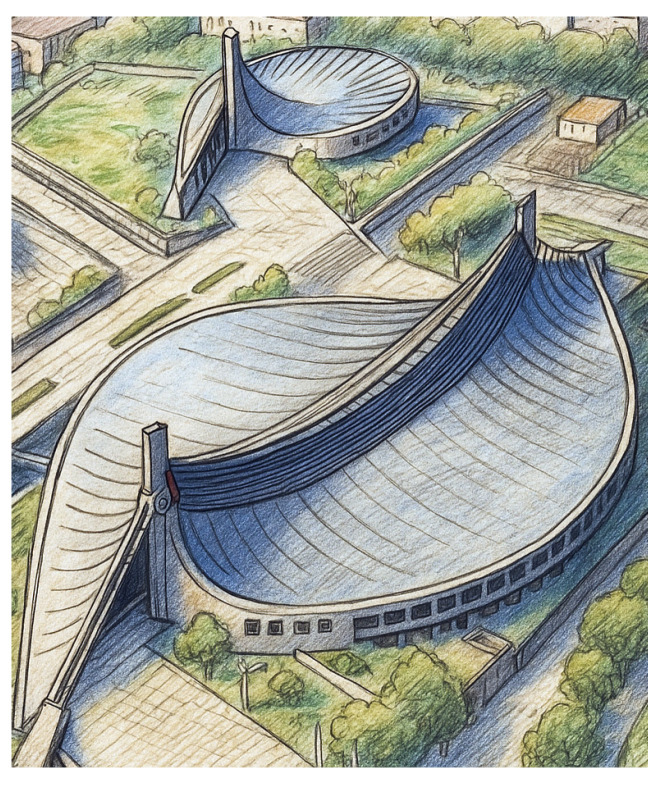
Drawing of the Tokyo Yoyogi National Gymnasium (author: Congrong Xiao).

Architectural structural biomimetics achieves the technological translation and structural innovation from natural prototypes to engineering applications through the systematic extraction of biomechanical principles. As a prototypical biomechanical system, the spider web—composed of radial main cables and spiral secondary cables—forms a lightweight and highly efficient dispersive network that provides an important bionic foundation for saddle-shaped cable-net structures in architecture. This bidirectional tension distribution mechanism is realized through the prestressed tensioning of steel cables, enabling large-span coverage while significantly reducing the self-weight of materials, thereby embodying the optimization principles of biological systems in structural efficiency. Suspension bridge technology has provided an essential engineering foundation for biomimetic structural design in architecture. The tower anchorage and cable deflection control techniques established in the 19th century by the Brooklyn Bridge served as technological precursors to the development of architectural suspension structures. However, biomimetic innovation in architecture does not entail a direct transplantation of bridge engineering methods; rather, it adapts the load-bearing characteristics of steel cables in conjunction with the multidirectional force distribution principles of spider webs to develop three-dimensional cable-net systems tailored to the specific requirements of buildings. This technological integration effectively addresses structural challenges unique to architecture, such as 3D surface stability and wind-induced vibration [8,13]. For example, the Tokyo Yoyogi National Gymnasium (Figure 5) exemplifies the successful architectural application of the saddle-shaped cable-net structure. In this project, the swimming arena employs two main cables anchored to tall pylons to form a crescent-shaped bidirectional surface, whereas the arena for ball games utilizes a spiral cable-net in combination with the compressive characteristics of shell structures to optimize nodal load distribution. The cable-net nodes draw upon the elastic connection mechanism of spider silk, with biomimetic algorithms employed to effectively mitigate stress concentration. Furthermore, the curved roof layout incorporates the leaf-vein light-guiding principle, enabling an organic integration of uniform natural light penetration and ventilation functions [15].

**Figure 6:**
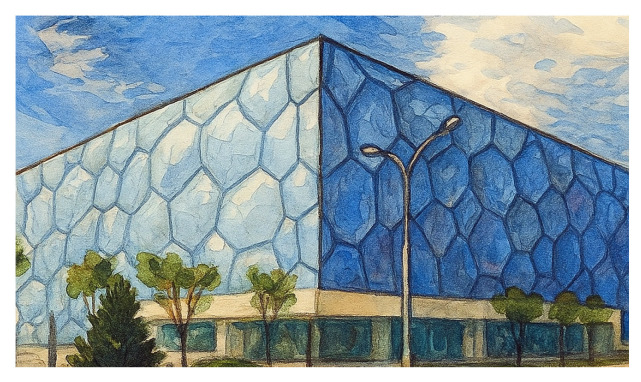
Drawing of the China National Swimming Center “Water Cube” (author: Congrong Xiao).

Another example is the China National Swimming Center “Water Cube” (Figure 6), where the ETFE membrane structure is directly inspired by the geometry of air bubbles. The curved unit system, composed of 3065 inflated air cushions, demonstrates significant advantages in terms of light transmittance, self-cleaning properties, and thermal insulation performance. Similarly, the ribbed concrete shell of the Palazzetto dello Sport in Rome embodies the application of biomimetic principles derived from eggshells in large-span structures. The thin-shell structure, with a diameter of 61 m, minimizes material usage while achieving large-span load-bearing capacity through the collaborative effect of 1620 prefabricated components and flying buttresses [15].

**Figure 7:**
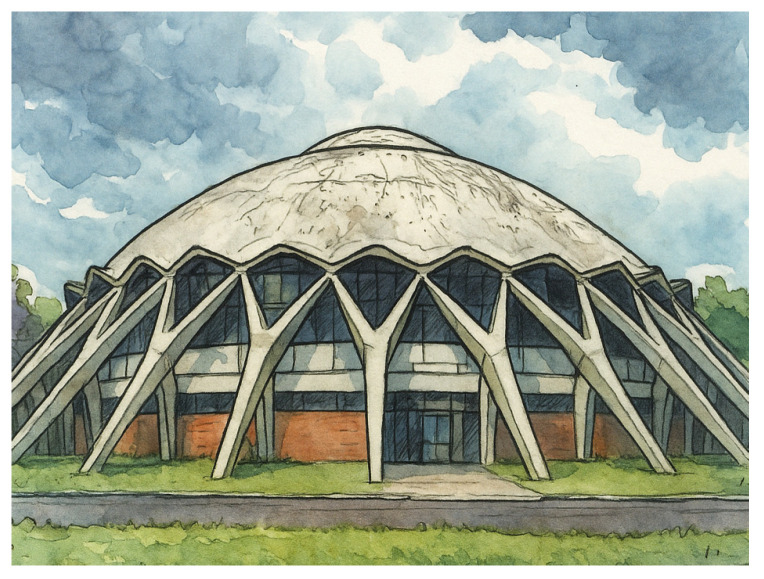
Drawing of the Palazzetto dello Sport (author: Congrong Xiao).

The biomimetic application of shell structures is likewise of critical significance. The ribbed concrete shell of the Rome Palazzetto dello Sport (Figure 7), spanning 61 m in diameter, is composed of prefabricated components and reinforced with flying buttresses. This thin-walled, eggshell-inspired form achieves material minimization and large-span load-bearing. Demonstrating minimal material usage, extensive spanning capacity, and robust durability, the structure underscores the technical advantages of structural biomimetics in realizing efficient building load-bearing and spatial innovation [29].

The design of biomimetic architectural structures must fully consider the structure-material coupling effect under various environmental conditions. The dynamic shape-adjusting ability of the prestressed cable-net system enables it to respond to seismic and strong wind loads, achieving an environmental adaptation mechanism similar to the elastic deformation observed in biological organisms. This biomimetic design concept not only enhances the structural safety performance but also reflects the harmonious unity between architecture and the natural environment, providing an important direction for the development of sustainable building technologies [13].

#### 3.2.2. Morphological Biomimetics of Architectural Design

Morphological bionics, through the systematic abstraction and refinement of biological characteristics, facilitates a profound expression of cultural metaphors, a feature particularly evident in contemporary museum architecture. This design methodology not only generates a unique architectural form language but also systematically optimizes architectural functions by mimicking the organizational principles found in nature. The core of morphological bionics lies in transforming the geometric logic of biological forms into organizational principles for architectural space, thereby establishing an organic connection between visual aesthetics and functional performance.

**Figure 8:**
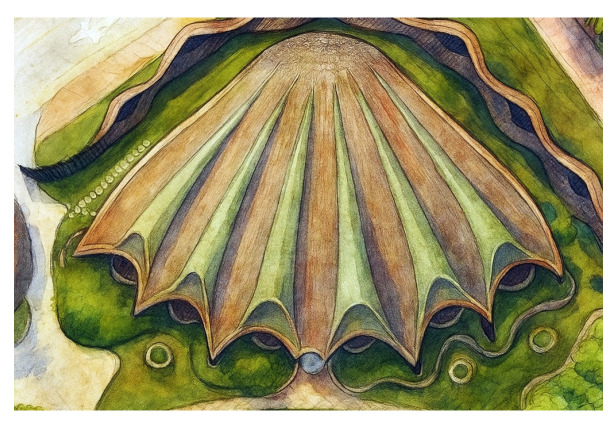
Drawing of the headquarters building of Shenzhen fashion brand Marisfrolg (author: Congrong Xiao).

The Shenzhen Marisfrolg Headquarters Building (Figure 8) exemplifies the integration of architectural design with morphological biomimetics, material biomimetics, and artistic design, using materials such as waste ceramic fragments, recycled bricks, and foamed ceramics to mimic the unique textures of natural elements like leaves, fish bones, and shells. Through the process of fragmentation and reconstruction, these materials form fluid, architectural curved surfaces. The entire building resembles a “fish leaping from the deep sea” or an “eagle spreading its wings,” with its internal structure ingeniously mimicking the interwoven form of mangrove root systems, ensuring biomechanical stability while creating a dynamic, fluid spatial experience. Particularly noteworthy is that the ceramic fragments used on the building’s exterior walls are sourced from recycled wine bottles, fully embodying the sustainable concept of the circular economy. Concurrently, the Shanghai Tower echoes natural tornadoes or DNA double helix structures with its spiral form, not only symbolizing the infinite continuation of vitality but also optimizing the building’s wind resistance at the engineering and technical levels, achieving a dual breakthrough in aesthetics and functionality.

**Figure 9:**
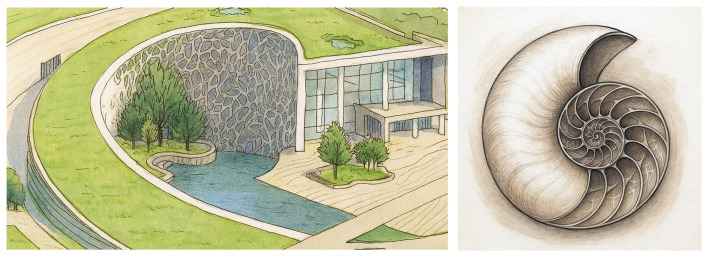
Drawing of the Shanghai Natural History Museum New Hall and Nautilus structure diagram (author: Congrong Xiao).

The Shanghai Natural History Museum New Hall (Figure 9) was jointly designed by the American architectural firm Perkins+Will and the Architectural Design and Research Institute of Tongji University. This project, inspired by the spiral geometric form of the nautilus shell, demonstrates a profound application of morphological bionics in cultural architecture. The design employs a spiraling curve that ascends, with a grass-slope landscape covering the roof and an elliptical pool at the center, collectively forming an ecological imagery system. The façade design adopts differentiated bionic skin strategies to achieve functional zoning: the “rock wall” on the northern façade metaphorically represents tectonic plate movements through linear textures, the vertical green wall on the eastern façade symbolizes Earth’s vegetation ecosystem, and the “cell wall” on the southern façade, characterized by a polygonal hollow structure, embodies the evolutionary process of life. This three-dimensional bionic design reflects the systematic translational capacity of curved surfaces as envelope enclosures, effectively conveying natural imagery [22].

**Figure 10:**
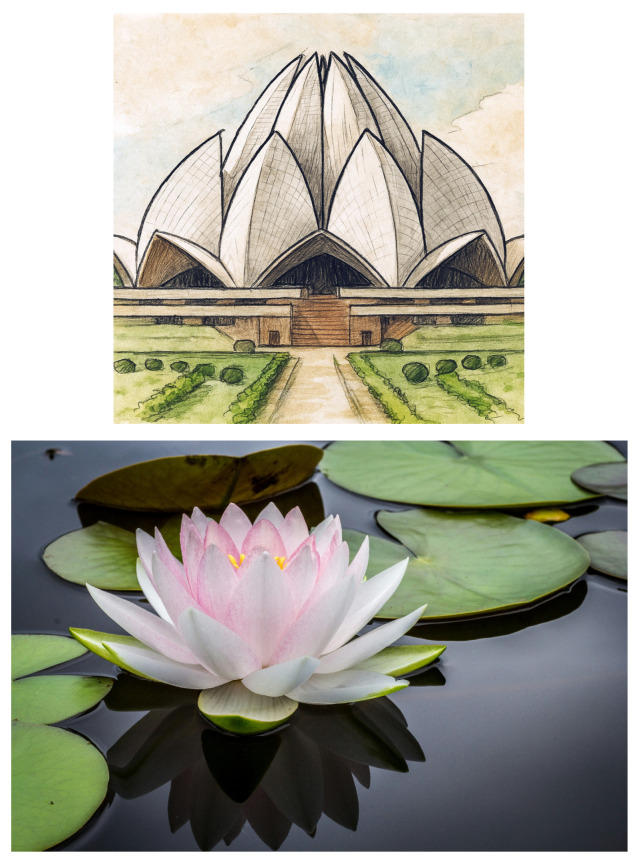
Drawing of the India Lotus Temple (author: Congrong Xiao) and a lotus (author: Jay Castor. source: unsplash.com).

However, morphological bionics must avoid superficiality. Excessive pursuit of visual form may detract from the architectural significance and should instead strive for cultural elevation through abstract bionic design. The petal reconstruction of the Indian Lotus Temple (Figure 10), consisting of 27 independent marble-clad “petals,” exemplifies the deep application of morphological bionics in cultural architecture. The building is composed of 27 separate marble “petals,” grouped in sets of three to form a nonagonal geometric configuration. Through precise surface reconstruction, it conveys the cultural meaning of religious purity. The design uses light and water as core decorative elements, replacing traditional statues and carvings found in Indian temples. Nine surrounding water pools create the visual effect of the building floating on water. This design strategy illustrates the technical logic of curved reflectors guiding natural light, achieving an organic integration of religious symbolism and spatial experience. Designed by Iranian-Canadian architect Fariborz Sahba, the temple draws inspiration from the lotus, symbolizing purity and beauty, and is surrounded by nine water pools that further enhance the floating visual impression [15].

#### 3.2.3. Functional Biomimetics of Architectural Design

Functional biomimetics emphasizes mimicking the functional characteristics of organisms, enabling buildings to possess adaptability and responsiveness similar to biological systems. In special environmental conditions, the design of biomimetic building skins is particularly important. Pinecone-inspired dynamic skin systems, for example, utilize novel adaptive materials to emulate pinecone scale responses to humidity conditions.

Pinecone scales possess unique dual-layer heterogeneous tissue structures, with the outer layer of hydrophobic lignified fibers and inner layer of hydrophilic cellulose tissue producing different degrees of expansion and contraction during humidity changes, creating stress differentials that drive scale opening and closing. This passive dynamic regulation mechanism requires no external energy input, relying entirely on moisture absorption differences in the materials themselves, providing a solid biological theoretical foundation for architectural biomimetic design [30].

The biomimetic building skin system successfully replicates the humidity response characteristics of pinecones through dual-layer composite material design and biomimetic hinge structures. The system employs a combination of hydrophilic wood layers and hydrophobic polymer layers, triggering deformation through humidity gradients and achieving intelligent opening and closing of skin units through combined control axis mechanical structures. When environmental humidity is high, the skin closes to reduce heat loss; when humidity is low, the skin opens to increase natural ventilation, effectively regulating indoor light and thermal environments, significantly enhancing building comfort while reducing energy consumption.

Despite promising applications, challenges remain in material durability, environmental adaptability, and cost scalability. With future developments in intelligent material technology and deeper multidisciplinary integration, the pinecone biomimetic skin system is expected to become one of the core technologies for green buildings, providing innovative solutions for building energy conservation and sustainable development. Currently, this technology has been validated in experimental projects at institutions such as Shanghai Jiao Tong University, demonstrating promising application potential.

**Figure 11:**
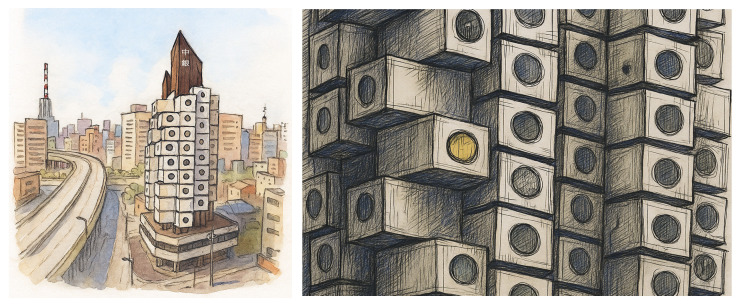
Drawing of the Nakagin Capsule Tower (author: Congrong Xiao).

Biological strain pattern biomimetics, including skin thermal response and metabolic structures, is another key area. For example, Kisho Kurokawa’s Nakagin Capsule Tower (Figure 11) embodies the design concept of architecture mimicking the adaptive evolution that organisms undergo in natural environments for survival. He decomposed the building into replaceable independent capsule units (140 total), with each capsule serving both residential and office functions, connected to water and electrical systems through a central core. When individual capsules age, they can be independently replaced, avoiding total demolition. Residents can customize or adjust capsule functions according to needs (such as office-residential conversion) [15].

#### 3.2.4. Summary of Biomimetic Art in Architectural Design

Comprehensive research indicates that the essence of biomimetic architecture lies in rooting human habitation within nature. Biomimetic technology not only provides new approaches and methods for architectural design but also points toward directions for sustainable architectural development. Future work requires deepening biomimetic research on biological strain patterns in technological transformation while avoiding symbolic collage, achieving architecture that integrates technology and art, thus reconstructing the symbiotic relationship between humans, buildings, and nature in an era of ecological crisis. Through in-depth research and application of biomimetic technology, architectural design will better serve human needs while harmoniously coexisting with the natural environment.

### 3.3. Biomimetic Art Design in Public Spaces

Biomimetic art design in public spaces serves as an essential component of people’s daily lives, enhancing spatial layering, visual richness, and emotional resonance. Through mimicking the forms, structures, and functional mechanisms found in nature, it effectively enhances the aesthetic value and functionality of spaces, offering the public a more natural and harmonious experience. As a vital carrier of urban culture, biomimetic art design not only strengthens the visual impact of spaces but also endows them with functionality, interactivity, and sustainability.

#### 3.3.1. Plant Biomimicry of Public Spaces

Plant biomimetic art design draws inspiration from the forms and growth patterns of natural plants. Through abstraction, biological characteristics are transformed into the language of public art. The integration of natural elements and the enhancement of spatial perception become core strategies of the design. Designers introduce plant forms or the structures of natural phenomena into their designs, effectively enhancing the spatial layering and visual richness [31].

**Figure 12:**
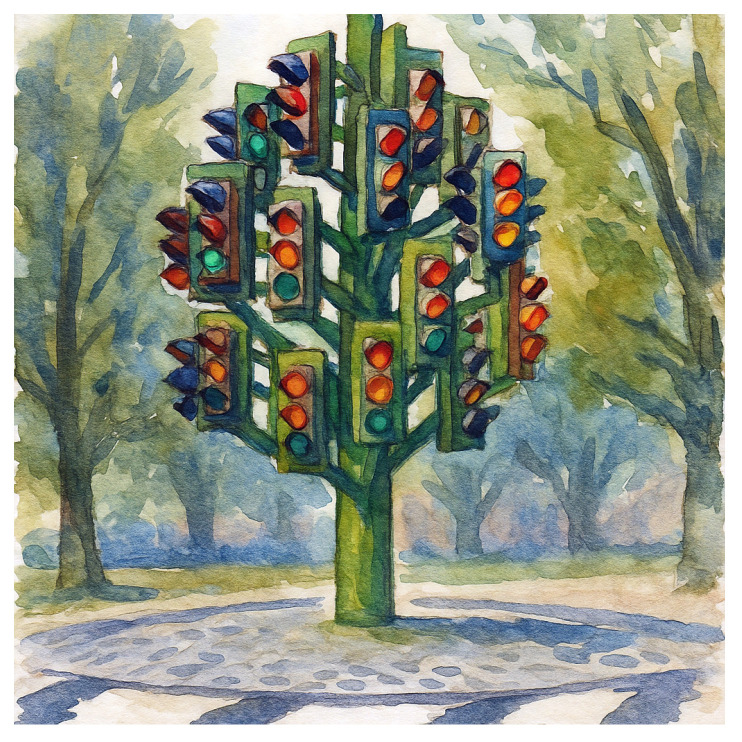
Drawing of the Traffic Light Tree (author: Congrong Xiao).

A typical example is the “Traffic Light Tree” in London, designed by French artist Pierre Vivant, as illustrated in Figure 12. Using the form of a tree as the prototype, over 70 sets of traffic lights are arranged to simulate the crown of a tree, mounted on an 8-m-high pole. The piece employs geometric curved surfaces, blending modern design aesthetics with biomimetic design, allowing the industrial traffic equipment to visually merge with the natural environment while addressing the abruptness of urban streets. This design not only incorporates characteristic properties and orderly forms but also merges the beauty of technology with that of nature, endowing public facilities with ecological functions [30].

**Figure 13:**
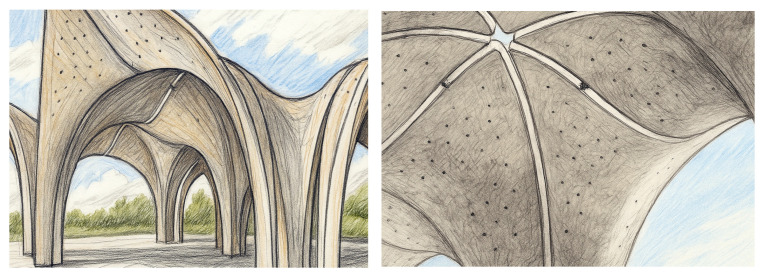
Drawing of the concrete petal sunshade pavilion in Confluence Park, USA (author: Congrong Xiao).

Additionally, the “Petal Sunshade Pavilion” at Confluence Park (Figure 13) serves as another example of form-based biomimetic design. The design cleverly mimics the water collection mechanism of plant leaves, utilizing carefully designed hyperbolic concrete petal structures to effectively direct natural rainfall toward the columned base. Through ingenious engineering design, water resources are channeled into an underground water storage system, providing a sustainable water recycling solution for arid regions. The 22 spirally arranged petals form a visually stunning dome structure, creating a dreamlike spatial effect with an interplay of light and shadow. The entire building possesses a strong sculptural presence while being imbued with ecological poetry, successfully fusing landscape art and functionality.

Plant biomimicry, through the gentle, soft, and dynamic characteristics of curved forms, weakens the mechanical feel of industrial products and establishes emotional bonds between humans and nature. It transforms public spaces into bridges connecting urban life and natural experiences, while simultaneously enhancing the sustainability of spaces through the utilization of natural energy conversion mechanisms.

#### 3.3.2. Animal Biomimicry of Public Spaces

Animal biomimicry focuses on the functional structures of animals, transforming their mechanical properties into design logic for architecture and installations. This design methodology creates facilities in public spaces that are both artistic and capable of evoking emotional resonance in observers by drawing upon the structural intelligence of animal forms.

**Figure 14:**
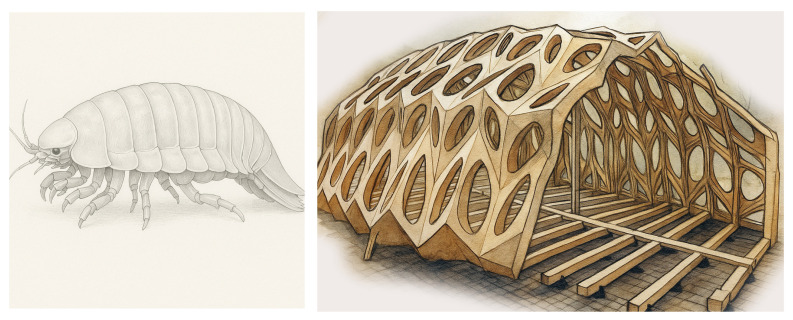
From left to right are replica images of the giant isopod and Bowoos temporary pavilion (author: Congrong Xiao).

The Bowoos temporary pavilion at Saarland University in Germany (Figure 14) draws inspiration from the exoskeleton of the giant isopod, using a wooden grid shell structure for material reduction and stability enhancement. Its uniform transitional characteristics of curved surface connections enable the building to possess both mechanical strength and biological affinity [30].

**Figure 15:**
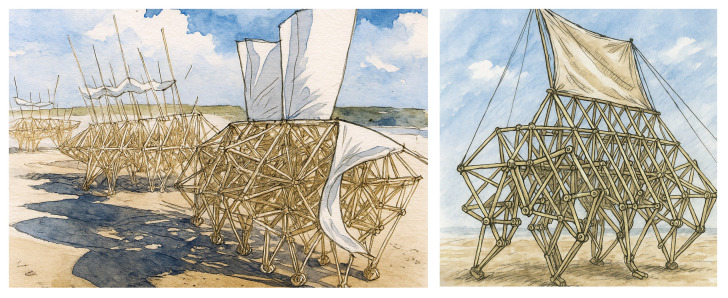
Drawing of STRANDBEESTS (author: Congrong Xiao).

**Figure 16:**
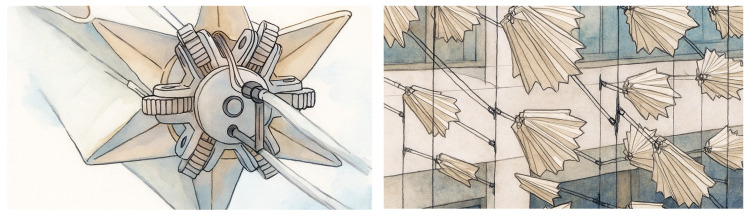
Drawing of Diffusion Choir (author: Congrong Xiao).

In the field of kinetic installations, Dutch artist Theo Jansen’s Strandbeest (Figure 15) stands as an iconic work. These giant installations composed of PVC tubes precisely mimic the walking mechanisms of insects, utilizing wind energy to drive complex articulated linkage systems to achieve autonomous locomotion on sand. It can even sense and automatically retreat from tides. The slow movement of these giant “biological colonies” along the coastline creates surreal kinetic sculptural landscapes, profoundly exploring the symbiosis between technology, nature, and humanity. Additionally, the “Diffusion Choir” installation by Sosolimited (Figure 16) consists of 400 umbrella-like units that mimic the opening and closing movements of jellyfish, utilizing flocking algorithms to control movements that simulate those of birds and fish schools, achieving coordinated dynamic transformations. The installation undulates with musical rhythms, creating wave-like audiovisual symphonies that inject immersive natural theater experiences into commercial space atriums, transforming the aesthetic qualities of biological behaviors into sensory art.

Animal biomimicry optimizes efficiency, safety, and aesthetics, leveraging evolutionary structural intelligence for public facilities.

#### 3.3.3. Biomimetic Materials of Public Spaces

Biomimetic design of materials plays a pivotal role in public spaces. By emulating natural material properties (such as the texture of stone and the grain patterns of wood) and even deeper structural principles (such as the efficient load-bearing capacity of bones and the hydrophobic functionality of skin) along with aesthetic principles (including form, color, and rhythm), designers can recreate the natural beauty and biophilic experiences within artificial environments, even endowing materials with functionalities that transcend their natural counterparts (such as self-cleaning, lightweight properties, and chromatic response capabilities).

Public facilities constructed from environmentally sustainable materials that simulate wood, stone, and other natural elements through modern technologies (such as 3D printing, digital modeling, and nano-modification) not only preserve the natural aesthetic essence of timber and stone materials but also, more critically, possess exceptional durability, low maintenance requirements, and superior environmental adaptability (including resistance to climate-induced erosion, inhibition of microbial growth, and slip-resistance properties). This significantly reduces consumption of natural resources and lowers lifecycle maintenance costs and ecological footprints, thereby embodying the core imperatives of sustainable development.

From a user experience perspective, the sophisticated application of biomimetic materials fulfills the biophilic needs of individuals in urban environments by providing “nature-like” tactile and visual experiences that align with human sensory preferences, contributing to enhanced spatial affinity and user well-being. Simultaneously, their customizable and enhanced performance characteristics (such as targeted improvements in slip-resistance, weather-resistance, or antimicrobial properties) provide support for safety and accessibility within public spaces.

This material application strategy transcends mere imitation; it represents a systematic design philosophy that integrates technology, ecology, aesthetics, and experience. It not only maintains the intrinsic spirit and value of natural aesthetics (through authentic or conceptual expression) but also effectively overcomes the numerous limitations of natural materials under conditions of intensive public use, complex climatic conditions, and resource constraints. It continuously propels public spaces toward enhanced functionality, more pleasurable user experiences, more economical maintenance, and greater environmental friendliness, harmoniously integrating functional, aesthetic, ecological, and social values, thereby creating healthier, more livable, and more attractive urban environments.

#### 3.3.4. Summary of Biomimetic Art Design in Public Spaces

Biomimetic design in public art design transcends mere replication of natural forms, creating works that meet human needs and possess both aesthetic value and functional requirements through understanding and applying the principles and wisdom of nature. Biomimetic art design in public spaces has evolved from singular morphological borrowing to systematic integration of “nature–technology–humanity.” Plant biomimetics reshapes urban aesthetics through visual integration, animal biomimetics transcends engineering limitations through structural optimization, while characteristic biomimetics responds to sustainable development imperatives through ecological wisdom. All three collectively point toward the core value of biomimetic design: establishing spiritual connections between humans and nature through users’ experiences with products, making public spaces material carriers of ecological civilization.

### 3.4. Biomimetic Expression in Fashion Design

Biomimetic design, as an innovative design concept, demonstrates distinctive artistic value and practical functionality in the field of fashion design. Through in-depth research and imitation of biological forms, structures, colors, and functions found in nature, fashion designers create garments that possess both natural aesthetic appeal and innovative spirit. Fashion design, as an important carrier of biomimetic art, unifies functionality and artistry by extracting core elements such as form, structure, color, and texture from natural organisms [17].

#### 3.4.1. Color and Form Biomimetics of Fashion Design

Color biomimetics enhances garments’ visual impact by imitating the color combinations of flora and fauna in nature. The color spectrum of natural organisms provides designers with rich inspiration, realized through the designer’s perception and understanding of color, representing an emotional fusion between the designer and nature.

**Figure 17:**
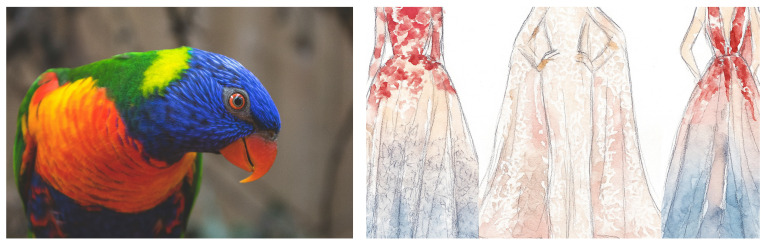
From left to right: rainbow finch (Author: Niklas Garnholz, Source: unsplash.com) and drawing from Elie Saab 2014 of gradient color garments (author: Congrong Xiao).

**Figure 18:**
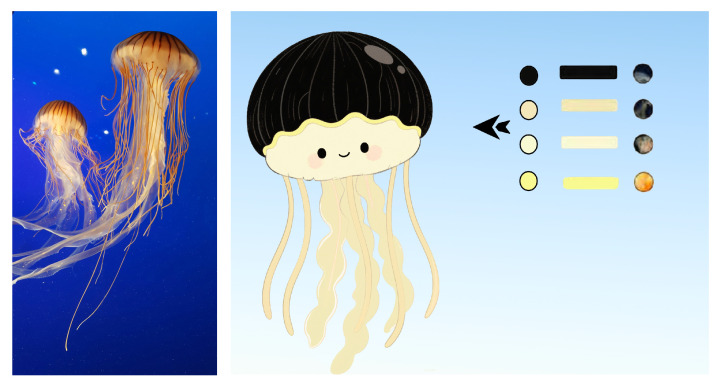
From left to right: a jellyfish from the deep sea (author: Taylor Boivin, source: unsplash.com) and a jellyfish illustration inspired by Li Qingxuan’s jellyfish color design (author: Congrong Xiao).

Designers draw inspiration from the abundant colors of the natural world, creating color combinations that are distinctly layered and full of vitality. For instance, the color distribution of the rainbow finch’s feathers has been directly applied to clothing color schemes, and Elie Saab’s 2014 Spring/Summer haute couture collection imitates the gradient hues of evening twilight (Figure 17). Additionally, Li Qingxuan created garments with distinct color layers and dynamic qualities by imitating jellyfish transparency and color gradation (Figure 18), using “goose yellow, light yellow, and black to form a visual contrast with the deep ocean environment.” [32]. This biomimetic color design not only enhances the aesthetic value of clothing but also demonstrates the dynamic beauty of nature through the fluidity and variability of color [27].

**Figure 19:**
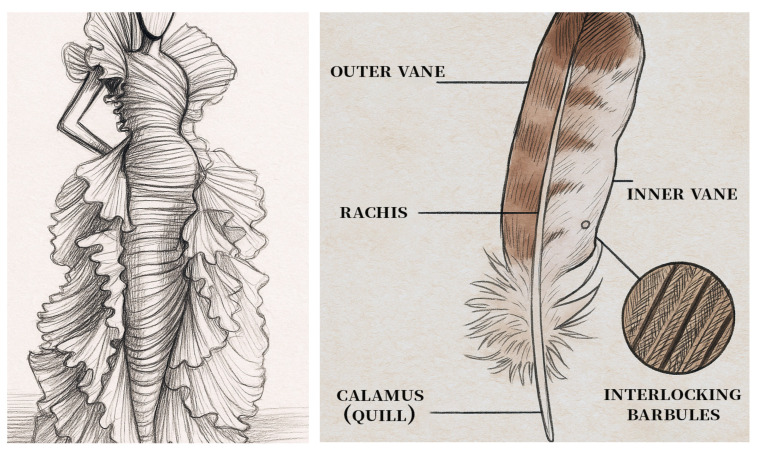
Drawing from Robert Wun 2023 and bird feather structure (author: Congrong Xiao).

**Figure 20:**
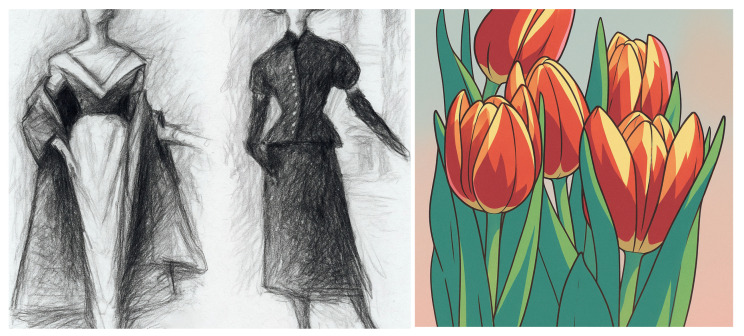
Drawing from Dior’s 1953 tulip series garments and of tulips (author: Congrong Xiao).

Robert Wun’s 2023 Spring/Summer collection (Figure 19) draws inspiration from bird wings, using 3D printing technology to “follow the arrangement and combination of feather structures, creating rhythmic spatial sensation.” [18] Additionally, Dior’s 1953 tulip series garments (Figure 20) skillfully integrated floral forms into fashion design, with “arched rounded shoulders, cinched waist, and elongated lower body, resembling the tulip stem in its entirety,” both showcasing natural beauty and reinforcing the feminine curves of the garments [17].

#### 3.4.2. Cultural Biomimetic of Fashion Design

Biomimetic design integrates natural wisdom with humanistic spirit, transcending mere formal imitation by not only embodying rich cultural connotations but also reflecting designers’ deep understanding and practice of sustainable development principles.

Sha Zenni’ Hug Me series combines floral and avian motifs from Qing dynasty embroidery with the modern theme of “embrace,” employing innovative temperature-sensitive yarn technology. When the cool-toned embroidered patterns come into contact with body temperature, they gradually transform into warm hues. This design not only metaphorically represents the transmission process of emotional warmth but also endows traditional cultural motifs with interactive characteristics of modern technology.

This design approach embodies the core concept of cultural biomimetic design: through artistic processing and technological upgrading of natural elements and cultural symbols, garments transcend their purely functional role to become three-dimensional media that carry cultural memory, convey emotional experiences, and express spiritual pursuits. While mimicking the temperature-sensing mechanisms of biological organisms in nature, designers simultaneously inherit and innovate traditional cultural expressions.

**Figure 21:**
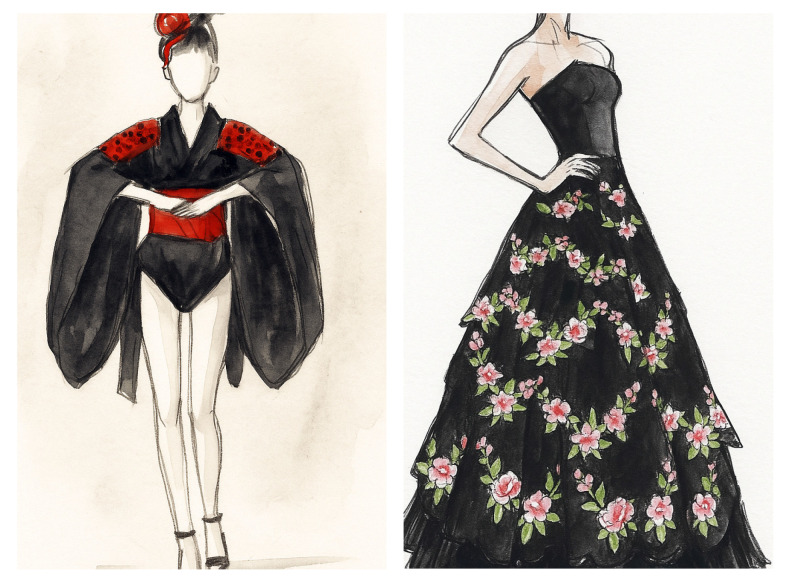
Drawing from NE·TIGER 2008 “Splendid National Colors: Chinese Formalwear” series (author: Congrong Xiao).

Another exemplary case is the 2008 NE·TIGER “Splendid National Colors: Chinese Formalwear” series (Figure 21). This design, grounded in profound cultural biomimetic principles, integrates millennia-old textile techniques such as Yunjin brocade and the Four Great Embroideries of China with the traditional Chinese color system of “national colors” accumulated over thousands of years. Through refined recombination of elements from Mawangdui silk paintings, Jinsha culture motifs, and the sartorial elements of over fifty ethnic groups, including Han, Tibetan, Miao, Dai, Yi, and Naxi, the designer constructs a decorative language that possesses both historical depth and contemporary dynamism. This process demonstrates the significant role of cultural biomimetic design in transmitting ethnic cultural DNA and innovatively transforming traditional cultural symbols.

In structural design, this series innovatively combines Western three-dimensional cutting techniques with the framework of Chinese ceremonial wear, employing a “skeletal membrane” structure to delineate Eastern body contours. While preserving the restrained elegance of traditional silhouettes such as cloud-shoulder collars, moonlight skirts, and pleated dry-skirts, it also incorporates the modern sculptural tension of demi-cup dresses, corseted bodices, pleated skirts, and fishtail gowns, presenting a flowing and graceful Eastern aesthetic that is both ethereal and exquisitely structured. This cross-cultural design integration exemplifies the positive significance of cultural biomimetic design in promoting intercultural exchange and dialogue between Eastern and Western cultures.

Regarding material application, the series innovatively employs multiple fabrics, including georgette, double-faced satin, Italian jacquard, lace, and Yunjin brocade, using biomimetic patchwork techniques to simulate the hierarchical textures of plant veins and feathers, complemented by Swarovski crystal light diffraction effects to create subtle variations in light, shadow, and tactile texture. This application of biomimetic technology not only enriches the visual layers of the garments but also establishes an organic connection between functionality and aesthetics, demonstrating the perfect fusion of modern craftsmanship and traditional cultural elements.

The application of cultural biomimetic design in fashion achieves the organic unity of aesthetic, functional, cultural, and ecological value. This design philosophy not only satisfies consumers’ pursuit of beauty but also strengthens ethnic cultural identity through the modernized expression of cultural symbols. Simultaneously, through the use of sustainable materials and the adoption of environmentally conscious craftsmanship, it reflects designers’ deep consideration and proactive action toward environmental protection. This multi-dimensional value manifestation positions cultural biomimetic design as an important bridge connecting tradition with modernity, nature with humanity, and aesthetics with responsibility [19].

#### 3.4.3. Structural Biomimetics of Fashion Design

Structural biomimetics in the realm of fashion design refers to the systematic study of internal structural principles found in biological organisms or natural materials—such as skeletal systems, nest architectures, and cellular arrangement patterns—and their transformation into garment support systems, silhouette shaping, and structural connections through abstraction and artistic design methodologies, thereby achieving functional optimization and morphological innovation in apparel.

**Figure 22:**
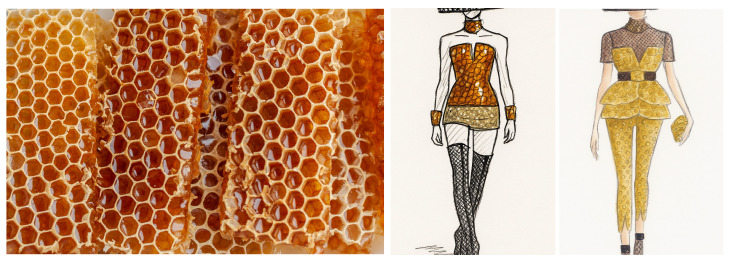
Honeycomb (author: Cristina Marin, source: unsplash.com) and drawing from the Alexander McQueen Spring/Summer 2013 Women’s Show (author: Congrong Xiao).

**Figure 23:**
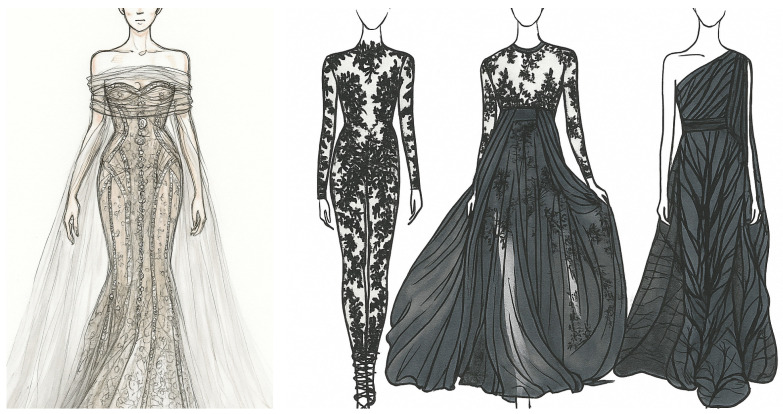
Drawing from the Zuhair Murad Fall/Winter 2013 Women’s Collection (author: Congrong Xiao).

In concrete design practice, structural biomimetics manifests through diversified applications and innovative cases. The sartorial application of honeycomb structures transforms hexagonal basic units into woven or assembled structural systems. The Alexander McQueen Spring/Summer 2013 collection (Figure 22) employed laser-cutting technology to precisely replicate dense hexagonal honeycomb patterns on leather materials, creating armor-like dress designs that combine protective functionality with breathability. The simulation of plant vine-wrapping structures mimics the curved coiling forms of climbing plants during their attachment and growth processes. The Zuhair Murad Fall/Winter 2013 collection (Figure 23) utilized metallic threads and bead embroidery to construct spiral vine texture systems across gown surfaces, with designs radiating outward from the core waist area to create dynamic growth visual effects.

**Figure 24:**
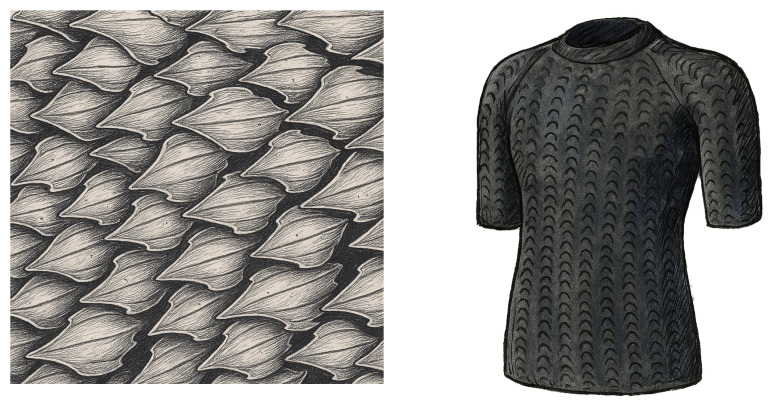
From left to right: draw of shark scale structure and draw of a shark-skin Swimsuit (Author: Congrong Xiao).

The development of shark-skin-like swimsuits (Figure 24) represents a classic application of structural biomimetics. Designers precisely mimicked the micro-denticle structures of shark skin to effectively reduce water drag and significantly enhance swimmers’ speed. During the 2000 Sydney Olympics swimming competition, Australian athlete Ian James Thorpe wore a black full-body wetsuit, resembling a shark advancing through blue waves, cutting through the water to win three gold medals. The shark-skin swimsuit he wore became renowned throughout the swimming world. Contemporary shark-skin-like swimsuits feature surface textures rendered through gradient laser printing technology, presenting captivating deep-sea-like luster that transforms purely competitive equipment into artistic objects bearing “oceanic aesthetic” symbols, embodying the unity of function and aesthetics [33].

Structural biomimetic design often relies on cutting-edge technological methods, including 3D printing technology, smart material applications, and parametric design approaches. 3D printing technology enables precise replication of complex biological structural morphologies (such as coral pore structures and spider web geometries), utilizing materials like photopolymer resin or nylon to fabricate lightweight wearable components. The development of smart materials, such as Bacillus subtilis natto-responsive fabrics that feature automatic opening and closing of ventilation pores when encountering perspiration, makes responsive functionality possible. Parametric design methods simulate biological growth and development patterns through algorithms, generating scalable structural pattern designs suitable for mass production.

### 3.5. Biomimetic Art in Product Design

Biomimetic practices in product design, as a significant design methodology, systematically simulate the forms, structures, functions, and cultural connotations of natural organisms, providing innovative approaches and sustainable development pathways for contemporary product design.

Biomimetic practices in contemporary product design primarily manifest as follows: form biomimicry creates products with aesthetic value by imitating the morphological characteristics of organisms in nature; function biomimicry enhances product practicality by drawing inspiration from biological physiological mechanisms and behavioral features; structural biomimicry optimizes the mechanical performance of products by studying the internal structures of natural organisms; and cultural biomimicry integrates the cultural significance of natural organisms into product design, achieving spiritual inheritance and innovation.

#### 3.5.1. Form Biomimicry of Product Design

Form biomimicry represents the most intuitive practice in product design, achieving visual aesthetics and emotional expression through concrete or abstract extraction of natural biological forms. This design methodology not only enhances the aesthetic value and functionality of products but also elevates the humanistic spiritual value embedded within them.

**Figure 25:**
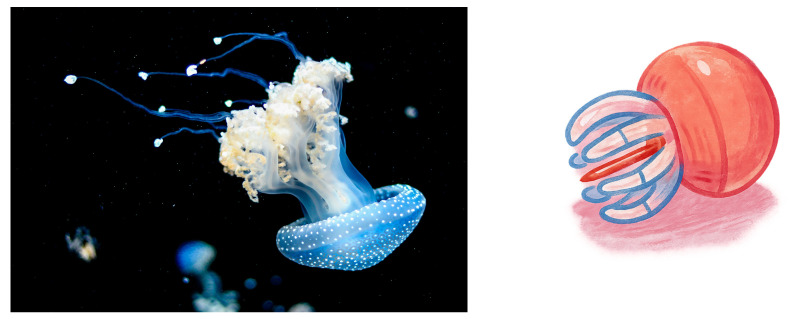
Jellyfish (Author: Tavis Beck, source: unsplash.com) and drawing from FVM ‘JELLYFISH’ PUSHPIN (Author: Congrong Xiao).

In specific design practices, the aforementioned flying fish-inspired drone (Figure 1) serves as a quintessential example. Additionally, the jellyfish pushpin designed, developed, and marketed by Fabio Verdelli Milano (Figure 25) ingeniously employs soft silicone material to simulate the morphological characteristics and elastic properties of jellyfish tentacles, achieving fixing functionality while preventing indentation damage to paper, and recreating the elegant swimming posture of jellyfish during compression. The Micro-Light Firefly jewelry collection utilizes fluorite as its core material to simulate the luminescent properties of fireflies, with packaging design that uniquely mimics the natural unfolding of leaves, creating a poetic opening experience for users.

#### 3.5.2. Function Biomimicry

Function biomimicry focuses on mimicking biological mechanisms to address technical challenges and optimize product performance. This design methodology translates functional principles from nature into technological features of products through in-depth investigation of physiological mechanisms.

**Figure 26:**
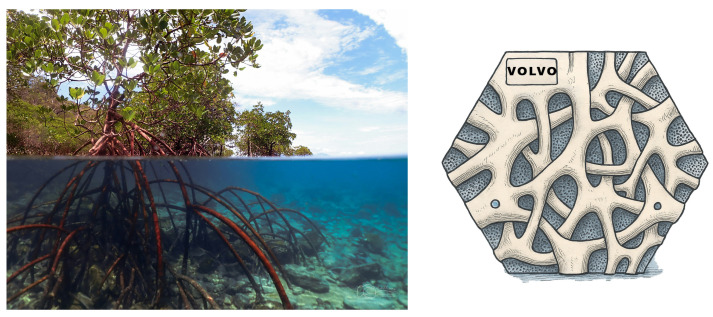
From left to right: Australian Mangroves (Author: Kristin Hoel, Source: unsplash.com) and drawing from the Volvo eco-friendly tiles project (Author: Congrong Xiao).

The Volvo Environmental Tile Project (Figure 26) demonstrates the application value of function biomimicry in sustainable design. This design simulates the interwoven root structure of Sydney’s native mangroves, with hexagonal forms mimicking the interlaced root structures of local Sydney mangroves, aimed at increasing the complexity of existing seawall structures and providing micro-structures for species that typically inhabit coastal areas, thereby compensating for biodiversity loss caused by traditional artificial coastlines. This practice demonstrates how function biomimicry transforms ecological wisdom into environmental solutions, thereby providing sustainable design solutions for coastal ecosystems [34].

#### 3.5.3. Structural Biomimicry of Product Design

Structural biomimetic design applies the structural principles of biological organisms (such as skeletal systems, honeycomb structures, leaf venation, etc.) to product design, achieving lightweight, high-strength, and functionally innovative solutions by optimizing mechanical distribution, material layout, and spatial configuration. This design approach particularly focuses on the mechanical properties of biological structures in nature and transforms them into structural solutions for product design.

**Figure 27:**
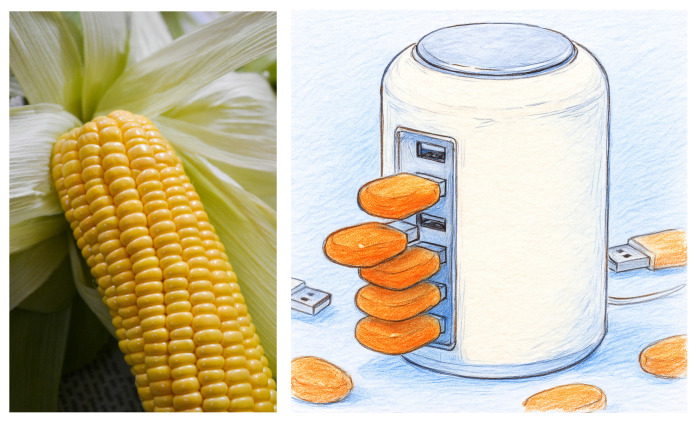
Corn (author: shraddha kulkarni, source: unsplash.com) and drawing of Ceramic Corn USB Drive (author: Congrong Xiao).

In addition to the aforementioned flying fish-inspired drone (Figure 1), the corn USB drive design (Figure 27) serves as an exemplary case, where the insertion and removal mechanism recreates the structural relationship between the corn cob and corn kernels. The corn USB drive’s form is deeply inspired by plant morphology. Its main body echoes the shape of individual corn kernels, thereby fulfilling data-storage functionality while imbuing the product with distinctive natural aesthetics and a playful character. Employing a typical biomimetic interlocking-structure approach, the design draws on the weak, snap-fit connection formed between kernels and the cob’s placenta tissue to enable modular insertion and removal. Specifically, each USB module (analogous to a single kernel) can be independently plugged into or detached from the base (analogous to the cob axis), while an array of receptacles on the base not only provides precise physical alignment but also establishes electrical contact with the storage units. This arrangement faithfully reproduces the mechanical logic of its biological prototype. Functionally, the design leverages the corn ear’s “separable on demand” trait: users may add or remove USB modules to flexibly adjust total storage capacity. This dynamic expandability transcends the static nature of conventional single-unit drives, offering a novel interactive experience in data storage. Thus, the emphasis lies not on replicating the exact connective strength found in nature, but on abstracting the detachable feature inherent to the weak kernel–axis interface. The core of this biomimetic strategy is the transplantation of the macroscopic kernel-to-cob embedding logic, rather than a literal duplication of its mechanical robustness.

**Figure 28:**
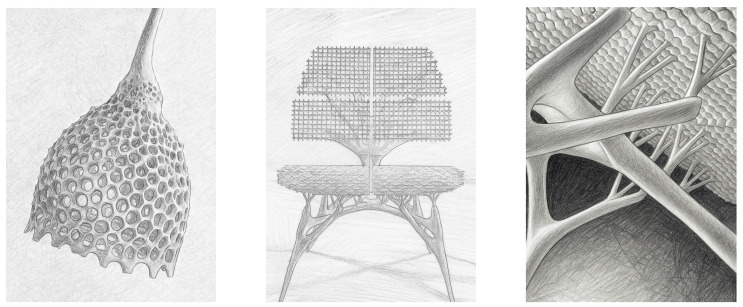
From left to right: draw of a certain Radiolarida (Author: Congrong Xiao) and drawing of a “Radiolaria #1” Life-size 3D Printed Chair Model (Author: Congrong Xiao).

Furthermore, the “Radiolaria #1” biomimetic chair design (Figure 28) launched by designer Lilian van Daal represents another quintessential case, demonstrating the profound integration of structural biomimicry with contemporary digital manufacturing technologies. This design takes marine planktonic unicellular organisms—radiolarians—as its prototype, achieving macroscopic translation of microscopic biological structures through 3D printing technology, transforming the radial symmetry characteristics of radiolarian skeletal structures found in nature into chair structures that exhibit elasticity, adaptability, robustness, and stability. The designer employed a single recycled polyamide material for fabrication, requiring no adhesive connections, thereby embodying the organic integration of sustainable design principles with circular economy thinking. This innovative practice not only achieves a 50% reduction in production time and energy consumption at the technical level but also demonstrates artistic interpretation of natural forms at the aesthetic level, reflecting contemporary designers’ design philosophy that integrates biological principles, environmental concepts, and artistic expression, providing significant theoretical reference and practical exemplars for the application of biomimetic design in the furniture manufacturing sector.

In the development of the self-cleaning function, designers have extensively drawn inspiration from the micro-nano dual-structure mechanism found on the surface of lotus leaves. The composite topological structure formed by micron-scale papillae and nano-scale waxy crystalline hairs on the lotus leaf surface achieves superhydrophobicity through the regulation of surface tension. By utilizing surface topology optimization techniques, designers have translated this biological principle into an engineering surface treatment solution, enabling self-cleaning and anti-fouling functions on material surfaces. This technology has been widely applied in fields such as building facades, textiles, and optical devices.

#### 3.5.4. Material Biomimicry of Product Design

Material biomimicry creates novel materials possessing high performance, sustainability, and intelligence through the imitation of biological materials’ structures, functions, or formation mechanisms. This approach transcends the performance boundaries of traditional materials, providing innovative pathways for product innovation. Its core principle lies in utilizing the wisdom of natural evolution accumulated over millions of years to optimize product performance, sustainability, and user experience.

The project “DIY Material Sustainable Design: Natural Resonance”, developed by product design students at Beijing University of Technology, demonstrates cutting-edge exploration in material biomimetic technology through thermochromic materials derived from wood chips and traditional Chinese medicine residues. Through 26 iterative experiments, the research team successfully combined recycled wood chips with traditional Chinese medicine residues, incorporating calcium alginate and gelatin adhesives to create innovative materials that simultaneously exhibit lightweight, antibacterial, and sound-absorbing properties, while endowing them with unique thermochromic functionality. This material has been ingeniously applied to screen and lighting fixture designs, where the screen automatically rotates and opens/closes according to temperature variations, while the “All Things Growing” themed lampshade transmits light through the natural textures of traditional Chinese medicine residues, projecting poetic natural patterns within the space, integrating functionality and artistry.

#### 3.5.5. Cultural Biomimicry of Product Design

Cultural biomimicry integrates cultural elements with biomimetic design thinking. It involves mimicking the structure, form, function, and symbolic meanings of natural objects, human-made objects, or symbols that carry cultural significance, thereby creating products that embody both cultural heritage and contemporary functionality. Through systematic exploration and transformation of cultural connotations, cultural biomimicry establishes a multi-dimensional application model encompassing superficial, intermediate, and deep layers, infusing modern product design with profound cultural foundations [6].

Cultural biomimetic design can be categorized into three levels based on the depth of cultural expression. Superficial cultural biomimicry primarily involves the direct extraction and application of the visual characteristics of natural organisms, including form, color, and texture, to convey specific cultural meanings [6]. This level is characterized by strong intuitiveness and high recognition value. One example is the design of the 2020 Tokyo Olympic torch, where the designer employed the natural form of cherry blossoms as a prototype, creating the cultural imagery of “the light of hope” through abstracted treatment of petal shapes, successfully combining the beautiful symbolism of cherry blossoms in Japanese traditional culture with the Olympic spirit. Intermediate cultural biomimicry focuses on simulating and transforming the behavioral characteristics of organisms, interpreting cultural customs and ritual connotations through behavioral pattern mapping, thereby creating richer cultural narrative experiences.

Deep cultural biomimicry expresses and communicates profound philosophical concepts and spiritual meanings through abstract atmospheric creation, exhibiting highly conceptual and spiritual characteristics. For instance, the design of the “High Mountain, Flowing Water” fragrance stand utilizes biomimetic abstraction of natural mountain and water forms, transforming the traditional Chinese cultural conception of “high mountains, flowing water” into concrete product morphology, conveying the Zen philosophical principles of “tranquility leading to distance, ethereal serenity”, achieving deep integration between material form and spiritual essence [6].

The cultural biomimetic design process requires the application of scientific methodological frameworks for systematic exploration and transformation of cultural connotations. During the product design transformation stage, cultural symbols are either directly imitated to preserve their original characteristics or innovatively interpreted through indirect transformation methods, realizing the contemporary expression of cultural values. Cultural biomimetic design not only preserves and promotes cultural traditions but also infuses modern product design with profound cultural foundations and spiritual meanings, achieving dual enhancement of both cultural and economic value.

#### 3.5.6. Color Biomimicry of Product Design

Color biomimicry design draws inspiration from biological colors in nature and transforms them into aesthetic and functional attributes for products. It transcends mere imitation of visual language, representing a technological reconstruction of the evolutionary logic of biological coloration (such as camouflage, signal transmission, and environmental adaptation) [12].

**Figure 29:**
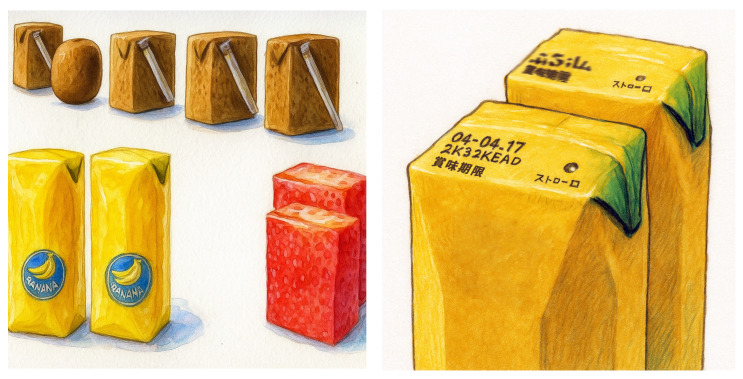
Banana (author: Giorgio Trovato, source: unsplash.com) and Kiwi (author: Pranjall Kumar, source: unsplash.com) and Drawing from “Juice Skin” by Naoto Fukasawa (author: Congrong Xiao).

In product packaging design, color biomimicry embodies the design principle of “form follows function.” For example, in the classic work “Juice Skin” series by Japanese designer Naoto Fukasawa (Figure 29), the banana beverage packaging adopts the bright yellow of bananas and simulates the texture and form of banana peels, enabling consumers to intuitively connect the product with natural fruit through visual and tactile perception without requiring textual instructions. This design technique utilizes pigment coloration principles to establish an intuitive connection between the product and natural food by mimicking the colors of biological skin, demonstrating the application value of color semiotics in biomimetic design.

**Figure 30:**
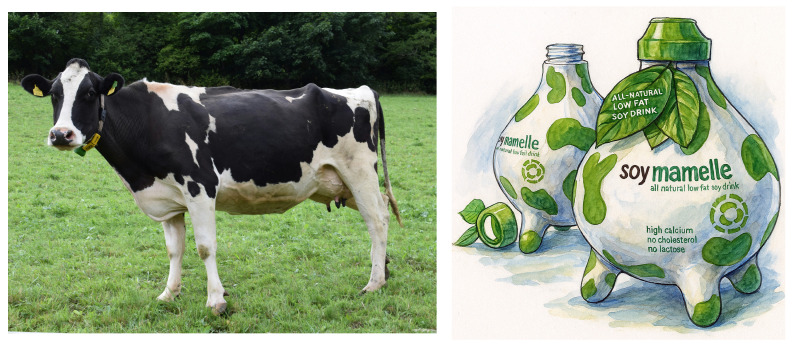
Dairy cow (author: Screenroad, source: unsplash.com) and Drawing of Russian milk packaging “Soy Mamelle” (author: Congrong Xiao).

The Russian milk packaging “Soy Mamelle” (Figure 30) draws inspiration from cow patterns, employing a three-color combination of black, white, and green, and utilizes latex materials to simulate biological tactile sensation, conveying a healthy and natural brand image. The innovation of this design lies in its integration of structural and color biomimicry to enhance product interactivity and playfulness through a squeezable packaging form.

**Figure 31:**
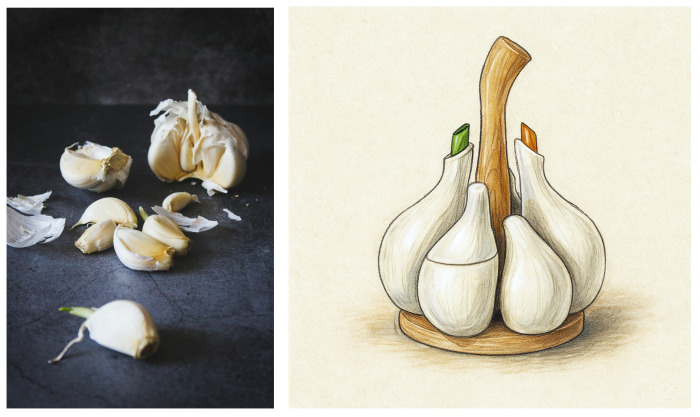
Garlic (Author: Tijana Drndarski, Source: unsplash.com) and drawing of Spanish Garlic Cruet “AJORí” (author: Congrong Xiao).

Similarly, the Spanish garlic seasoning bottle “AJORí” (Figure 31) replicates the purple-white gradient and layered structure of garlic cloves. It uses ceramic materials that reproduce natural texture to enhance aesthetic value while maintaining high recognition.

#### 3.5.7. Summary of Biomimetic Art in Product Design

Biomimetic product design has evolved from simple imitation of natural forms to a more comprehensive methodology, exhibiting developmental characteristics that transition from singular morphological imitation toward multidimensional integrated innovation, from the pursuit of superficial visual effects to in-depth learning of functional mechanisms, and from partial design elements to holistic design systems. By integrating biomimetic morphology and extended innovation methods, designers can generate creative concepts for new products, thereby broadening the role of biomimetics in product design.

## 4. Development Trends and Innovation Directions in Bionic Art Design

Biomimetic art design is undergoing a profound transformation from morphological imitation to systematic integration. Traditional biomimetic design primarily focused on simulating apparent characteristics such as form and color, whereas contemporary trends are shifting toward multidisciplinary, collaborative, and systematic integration models. Biomimetics has deeply intersected with disciplines such as mathematics, biology, and materials science, forming specialized branches such as “biomimetic mathematics,” “biomimetic electronics,” and “biomimetic art.”

### 4.1. Construction of Ecological Biomimetic Systems Oriented Toward Sustainable Development

Ecological biomimetic design promotes the development of sustainable engineering systems through circular self-organization. Its core lies in drawing inspiration from nature’s efficient material and energy cycles—from biomimetic materials at the molecular level to systematic symbiosis at the urban scale—achieving “waste-free” production and full life-cycle closed-loop management. This paradigm has transcended superficial imitation of natural forms, shifting toward multi-scale, multi-hierarchical ecological system integration. Chinese designers must not only adhere to environmental risk assessments stipulated by GB/T 42444-2023 (GB/T 42444-2023 Biomimetics: Terminology, Concepts and Methodology is a standard issued by the Standardization Administration of China. This standard aims to systematically organize and standardize terminology, concepts, and methodology in the field of biomimetics, providing a unified foundation for research, application, and communication in related fields) but also avoid secondary disturbance to ecosystems during the innovation process, ensuring biomimetic technologies resonate harmoniously with nature. GB/T 42444-2023 requires designers to implement environmental risk assessments in material selection, morphological biomimicry, and end-of-life cycle phases, incorporating considerations of cultural and ecological sensitivity, such as avoiding the use of fungal materials in specific religious domains, or guiding user participation in ecological restoration through seed paper packaging. In morphological biomimicry, it is also necessary to avoid interfering with wildlife behavior, such as avoiding bird courtship color frequencies when developing chameleon-inspired biomimetic skins.

In the field of material innovation, biomimetic biodegradable materials form the foundation of ecological biomimetic systems. Different types of biomimetic materials, including fungal mycelium packaging, spider silk protein sutures, and mollusk biomineralization layers, each possess distinct advantages and limitations. Biodegradable packaging inspired by fungal mycelium has achieved commercialization. In addition, spider silk proteins are gaining prominence in the medical field due to their ultra-high strength, while biomimetic nacre remains difficult to produce at scale due to high energy consumption and costs, despite possessing superior structural properties. Future research is needed to improve degradation controllability, reduce manufacturing costs, and reveal industrialization pathways.

In the field of fashion, contemporary designers actively adopt renewable materials and environmentally friendly processes in their creative endeavors, addressing the urgent demands of contemporary society for ecological environmental protection while also demonstrating a commitment to social responsibility. Through in-depth research into plant growth mechanisms and the ecological circulation patterns of the natural world, designers have developed various bio-based materials and biodegradable, eco-friendly fabrics. These innovative materials not only meet the fundamental requirements of modern clothing in terms of functionality but also achieve significant advancements in environmental friendliness. Novel bio-based fibers developed through mimicking plant fiber structures (such as polylactic acid fibers extracted from corn and sugarcane, as well as bio-based materials that imitate plant fiber structures, including abaca and bamboo fibers) maintain excellent wearing comfort while possessing fully biodegradable characteristics, fully realizing the design philosophy of “from nature, returning to nature.” [17].

In energy conversion and environmental resource utilization, ecological biomimetic design has likewise demonstrated numerous practical applications. Building photovoltaic skins based on photosynthesis principles (such as the Munich BIO facade) and artificial photosynthesis devices are making energy conversion processes “visible,” while fog-harvesting technology inspired by desert beetle water collection principles has achieved daily water production of approximately 10 L per square meter in arid regions, though maintenance costs and cultural adaptability require optimization. The systematic symbiosis concept further integrates waste-energy-material systems: although “sponge city” and industrial ecology park cases each have distinct focuses, they all embody cross-disciplinary, multi-scale cascaded utilization approaches [30].

Art design plays a triple role in ecological biomimetic systems, connecting technology with the public, and nature with cities. First, it transforms cold biomimetic materials into tangible narrative symbols—such as packaging surfaces that preserve the natural texture of mycelium, or mineralization layers that simulate pearl luster through laser etching; second, artists integrate technical facilities such as photovoltaic facades, fog-harvesting devices, and rainwater retention systems into cultural contexts through scenario-based design, such as “atrapanieblas” fog nets with Andean weaving patterns and the ecological corridor landscape transformation at Beijing Olympic Park; finally, artistic intervention provides spatial narratives for urban-scale systems, combining energy flow, material circulation, and dynamic data visualization to shape embodied “industrial metabolic atlases.”

Looking forward, the sustainable development pathway of ecological biomimetic design must rely on interdisciplinary collaboration and policy support. To bridge the gap between laboratory and industrialization, designers, biologists, materials scientists, and urban planners should jointly establish transparent energy efficiency and environmental impact indicators; simultaneously, innovative mechanisms such as carbon trading systems and biomimetic materials community craft workshops can be leveraged to achieve true closed loops from technological efficiency to social acceptance. Only within the triangular framework of “policy–science–art” can we advance toward a biomimetic paradigm of “ecology art” symbiosis, providing viable and sustainable solutions for addressing global environmental challenges.

### 4.2. Technological Innovation and Interdisciplinary Development

With continuous technological advancement, biomimetic art design demonstrates tremendous innovative potential in technical applications. Increased understanding of biological materials and electronic systems is expanding the capabilities of this field, enabling design works not only to imitate natural forms but also to achieve functional breakthroughs. The progress in biomimetic design relies on the development of multidisciplinary knowledge networks. For example, at the basic research level, biologists decode the mechanical advantages of hexagonal honeycomb structures, which engineers then transform and apply to aircraft honeycomb composite panels; neuroscientists explore octopus arm movement algorithms, providing control models for soft robotics. At the technological transformation level, a three-tier research and development system of “biological prototype–technical deconstruction–product realization” is established. For instance, antibacterial materials based on shark skin microstructures, optimized through fluid dynamics, are applied to biomimetic swimwear and antibacterial surface treatments for medical devices. Interdisciplinary collaboration not only resolves technical isolation problems but also catalyzes emerging professions such as biological architects and biomimetic materials engineers, driving a paradigm shift across industries [35].

At the digital technology level, artificial intelligence and algorithmic design serve as core tools by simulating biological intelligent systems through neural networks to achieve adaptive morphological generation; utilizing ant colony algorithms to optimize product structural pathways and enhance functional efficiency; and combining evolutionary algorithms to drive iterative evolution of biomimetic forms, such as product morphological derivation models based on principles of genetic mutation [5].

Biomimetic research in the social sciences further expands design dimensions. Future innovative development requires dismantling disciplinary barriers and constructing a tripart integration model of “natural sciences–social sciences–art design” to transition from singular morphological biomimicry to ecosystem biomimicry. Therefore, biomimetic art design demonstrates clear interdisciplinary development trends, increasingly emerging as a comprehensive interdisciplinary field that integrates knowledge systems from art, engineering, biology, and other disciplines. This interdisciplinary fusion not only broadens design perspectives but also provides greater possibilities for innovation.

The development trends of biomimetic art design are also evident in the enrichment of cultural connotations and the deepening of humanistic emotional dimensions. Modern biomimetic design no longer pursues mere functionality but integrates cultural elements, making it not only functional but also rich in symbolic meaning and emotional appeal. This emotional value creation manifests in three major transitions: natural sentiment awakening, life dynamic resonance, and cultural gene translation. French designer Philippe Starck’s branch-form faucet uses inverted tree branch morphology to evoke users’ subconscious associations with forests, transforming the daily act of handwashing into a poetic experience; the Exocet Chair mimics the dynamic curves of fish tails through wooden slats, allowing users to achieve different resting postures by adjusting angles, experiencing life rhythms through interaction. Chinese designers combine “bamboo node growth” morphology with traditional mortise and tenon craftsmanship, creating furniture products that conform to mechanical structures while embodying Eastern philosophy. Such designs construct spiritual healing spaces beyond the functional level through biological metaphors and emotional interaction mechanisms, responding to modern people’s psychological need to connect with nature [24].

In summary, the development trends of biomimetic art design exhibit diversified and integrated characteristics, with prospects presenting multi-dimensional fusion patterns. The dissolution of disciplinary barriers between art, engineering, and biology will stimulate innovation, while development consistently revolves around two core propositions: how technology can more deeply reveal and apply life wisdom, and how design can promote human–nature symbiosis [35].

By mimicking the operational mechanisms of natural ecosystems, biomimetic art design will increasingly influence works that are both aesthetically pleasing and environmentally friendly. Future designers need to develop a deeper understanding of the morphological structures and patterns of natural phenomena, integrating ecological thinking into every aspect of design to achieve harmonious unity between artificial and natural environments. Only by infusing ecological ethics and humanistic care into technological innovation can biomimetic design truly bridge civilization and nature, providing richer theoretical guidance and practical pathways for future design practice.

## 5. Conclusions

Biomimetic Design, emerging in the 1960s with the disciplinary formation of Bionics, has undergone a paradigm shift from a mere focus on Morphological Imitation toward a Systemic Approach. Early investigations primarily concentrated on the external replication of biological forms and structures, whereas contemporary biomimetic design places greater emphasis on in-depth analysis of the multi-layered characteristics of biological systems. These include morphological attributes (e.g., the hydrodynamic properties of sharks), structural principles (e.g., the lightweight advantage of honeycomb configurations), functional mechanisms (e.g., the humidity-responsive behavior of pinecones), and ecological self-organization patterns, which are subsequently transformed into cross-disciplinary design strategies. Bibliometric evidence indicates that although biomimetic research has expanded from mechanical engineering into the realms of art and design, systematic theoretical development addressing the philosophical and artistic dimensions of biomimesis remains relatively underdeveloped, accounting for only 3.9% of the total scholarly output.

In the fields of architecture and urban design, Structural Biomimetics and Ecological Biomimetics have emerged as significant strategies. For instance, the suspension structure of the Yoyogi National Gymnasium in Tokyo draws inspiration from the mechanical distribution principles of spider webs, while the ETFE membrane structure of the Water Cube in Beijing is inspired by the form of soap bubbles, achieving both structural lightness and high transparency. In the field of product design, Functional Biomimetics has led to numerous innovations. For example, the Shinkansen train is modeled after the streamlined shape of the kingfisher to reduce air resistance, and Shark-Skin Swimwear utilizes microscopic surface textures to reduce drag, enhancing swimming efficiency. Meanwhile, Cultural Biomimicry goes beyond mere visual imitation, merging traditional cultural symbols with natural forms. This approach not only elevates the aesthetic value and emotional resonance of design works but also imbues them with profound socio-cultural significance, facilitating the innovative transformation of traditional culture in a modern context. In the materials domain, emerging Biomimetic Materials exhibit vast application potential. For example, self-healing concrete inspired by shell structures and biodegradable packaging materials derived from fungal mycelium are advancing the realization of material circularity through the integration of parametric design and 3D printing technologies. With the development of Intelligent Responsive Technology, biomimetic design is advancing towards adaptive and intelligent solutions. For instance, a pinecone-inspired facade employs thermally responsive materials for dynamic opening and closing, while chameleon biomimetics utilizes a color-changing system to respond to environmental conditions.

At the ecosystem level, biomimetic design has evolved from simple morphological imitation to the development of Ecological Biomimetic Systems. The Living Water Park in Chengdu mimics the self-purification mechanism of wetlands, creating an ecological chain comprising anaerobic ponds, plant beds, and fish ponds to achieve the systematic integration of sustainable design. Similarly, the concept of the Sponge City has transformed urban rainwater management through the simulation of natural hydrological cycles. These practices demonstrate that biomimetic design is becoming a crucial strategy in addressing ecological crises and promoting sustainable development.

Biomimetic Design has evolved from superficial biological form replication into a systemic innovation paradigm that integrates function, structure, ecology, and cultural values. Through the conversion of multi-scale natural intelligence—ranging from the microscopic layered structures of shells to the macroscopic urban hydrological cycles—biomimetic design has not only achieved advancements in visual aesthetics and functional optimization but also redefined the symbiotic relationship between humans, nature, and technology in addressing the challenges of sustainable development. Looking ahead, deepening interdisciplinary collaboration between biology, materials science, and digital technologies, coupled with an ethics-driven technological pathway, will be instrumental in realizing the ultimate vision of ecological aesthetics.

## Data Availability

The original contributions presented in this study are included in the article. Further inquiries can be directed to the corresponding author.
